# Computational evaluation of chromone–tetrazole hybrids as noncovalent inhibitors of SARS-CoV-2 M^pro^

**DOI:** 10.1371/journal.pone.0362385

**Published:** 2026-11-11

**Authors:** Oussama Khibech, Salma Kadda, Yousra Hammouti, Said Abadi, Shehdeh Jodeh, Belkheir Hammouti, Allal Challioui

**Affiliations:** 1 University Mohammed Premier, Faculty of Science, Laboratory of Applied Chemistry and Environment (LCAE), Oujda, Morocco; 2 Euro-Mediterranean University of Fes, UEMF, Fez, Morocco; 3 BIOPI–BioEcoAgro Joint Research Unit 1158, INRAE, University of Picardie Jules Verne, Amiens, France; 4 Department of Chemistry, An-Najah National University, Nablus, Palestine; Vellore Institute of Technology, INDIA

## Abstract

Emerging SARS-CoV-2 variants continue to demand orally tractable, non-covalent inhibitors of the conserved main protease (M^pro^). Here, chromone-phenyl-tetrazole hybrids were prioritized using a multiscale computational strategy that connects drug-likeness, binding stability, free-energy ranking, conformational behavior, and quantum-level electronic features. The series showed favorable oral drug-like profiles and low-to-moderate predicted acute toxicity. Among the stable M^pro^ complexes, M2 displayed the strongest computed affinity (MM/GBSA ΔG_bind_ = −38.8 kcal·mol ⁻ ¹), driven mainly by dispersion with limited desolvation cost, whereas M4 showed the most direct catalytic-dyad engagement through His41/Cys145-supported anchoring. DFT analysis rationalized these complementary profiles: M2 and M4 exhibited narrower HOMO-LUMO gaps (4.00 eV) and higher electronic softness, while ELF/NCI maps revealed heteroatom-centered electron reservoirs and weak intramolecular preorganization. The derived pharmacophore consists of a planar chromone π-platform bearing a carbonyl/phenolic acceptor-donor edge, a para-phenyl spacer for conjugation and hydrophobic occupancy, and a methoxy-tetrazole polar/π-rich head for Glu166/Phe140/His163 anchoring. Overall, M2 and M4 emerge as complementary lead chemotypes for experimental validation and structure-guided optimization against M^pro^.

## 1. Introduction

Since the onset of the coronavirus pandemic, medicinal‑chemistry research has shifted rapidly toward small‑molecule strategies that bypass the genetic drift affecting spike‑targeted antibodies [1]. The main protease of SARS‑CoV‑two has emerged as the most widely studied enzymatic target because its catalytic cysteine-histidine dyad is indispensable for polyprotein processing and has no close analogue in human biology, minimizing concerns of off‑target toxicity [2,3]. Early efforts concentrated on peptidomimetic inhibitors that borrow electrophilic warheads from work on retroviral proteases; while these covalent agents demonstrated impressive potency, their highly polar backbones and pronounced conformational freedom translated into modest oral exposure and a need for pharmacokinetic enhancers [4,5]. In response, the literature has pivoted toward rigid heteroaromatic scaffolds capable of engaging the catalytic dyad through non‑covalent clamps while maintaining the lipophilicity-polarity balance required for passive intestinal uptake [6,7]. Flavone [8], indole [9,10], benzotriazole [11] and thiazole [12,13] frameworks have all been explored in this context, yet the chromone skeleton remains particularly attractive because its bicyclic plane offers two well‑positioned hydrogen‑bond acceptors that can clip the oxyanion hole without resorting to reactive chemistry [14]. Parallel crystallographic campaigns have underscored the importance of the glutamate residue (Glu166) that gates the S‑one pocket, revealing that ligands able to interlock this residue together with the histidine partner (His41) of the catalytic dyad can freeze the protease in a catalytically incompetent conformation [6]. Complementary reviews on absorption, distribution, metabolism and excretion emphasize that nonpeptidic inhibitors must satisfy the dual requirement of moderate polarity and restrained rotatable bond count to reach systemic circulation without metabolic overload [15–18]. Within this design space, the tetrazole ring has gained favor as a bio‑isosteric acid that can replace carboxylates, reinforce hydrogen bonding to cysteine‑rich environments through π‑sulfur contacts, and tune overall polarity through targeted hydroxyl substitution on adjacent aromatic rings [19,20]. Recent computational pipelines therefore integrate multiplatform predictors of drug-likeness, pharmacokinetics, and toxicity SwissADME, pkCSM, ADMETlab 3, and ProTox-III in tandem with rigorous docking validated against high-resolution crystal structures and extended all-atom molecular-dynamics simulations executed in modern engines such as GROMACS.These multi‑layered approaches converge on the principle that synthetically economical, semi‑rigid heteroaromatics with finely adjusted polar surfaces can match or exceed the binding efficiency of bulkier covalent probes while offering superior oral developability and a broader safety margin.

Against this backdrop, the present study employs a fully integrated in‑silico workflow to evaluate a set of chromone-tetrazole hybrids (Scheme 1) as prospective non‑covalent inhibitors of the coronavirus main protease, laying the groundwork for experimental validation and future lead optimisation.

**Scheme 1:**
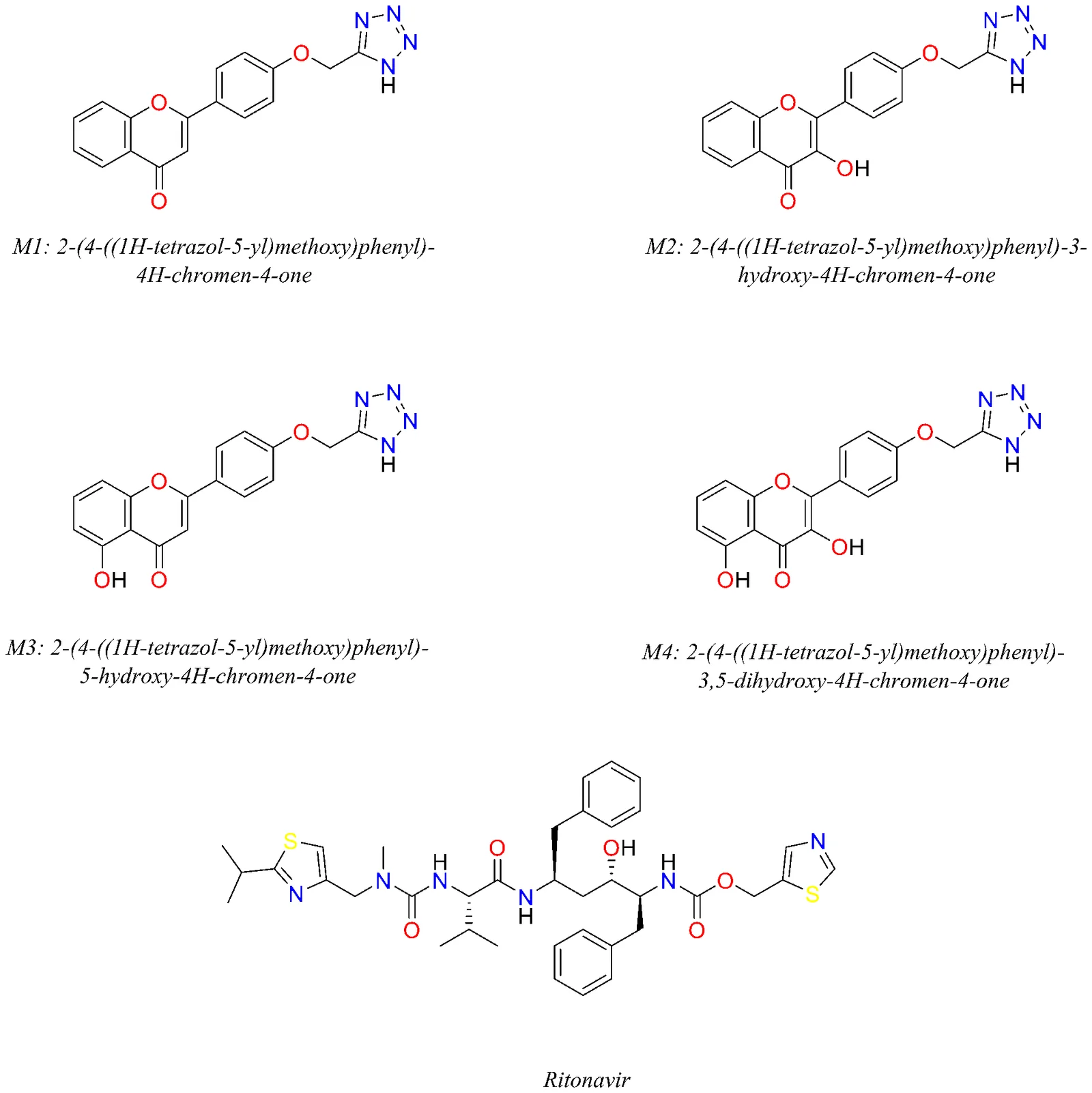
Molecular structures of the chromone-tetrazole derivatives M1-M4 and the reference inhibitor ritonavir.

The chromone-tetrazole series was selected on the basis of a prospective selectivity hypothesis rather than demonstrated selectivity. M^pro^ presents a conserved S1/oxyanion region and S2 hydrophobic pocket organized around the His41-Cys145 catalytic dyad [2,3]. The chromone carbonyl/phenolic edge was intended to address the polar S1/oxyanion network, the para-phenyl spacer to occupy the hydrophobic channel, and the tetrazole terminus to provide compact polar anchoring [19,20]. Avoiding an electrophilic warhead may reduce nonspecific thiol reactivity, while the compact semi-rigid scaffold may limit promiscuous conformational adaptation. Nevertheless, selectivity over human cysteine and serine proteases was not assessed; all selectivity statements are therefore presented as design rationale and require experimental counter-screening.

## 2. Materials and methods

### 2.1 Pharmacokinetic analysis using computational tools

The pharmacokinetic profile of a molecule its absorption, distribution, metabolism and excretion (ADME) is shaped by a succession of biochemical and physiological processes that govern how the compound traverses biological membranes, disperses through the circulation, undergoes biotransformation and is ultimately eliminated. A comprehensive understanding of these factors is critical for anticipating a drug’s efficacy and safety. Computational methods now play a central role in this assessment, offering rapid, chemistry‑based predictions of membrane permeability, target interactions and metabolic stability.

Each candidate molecule was sketched in ChemDraw, converted to a SMILES string, and evaluated on three complementary web platforms SwissADME [21], pkCSM [22], and ADMETlab3 [23]. SwissADME provided a first-pass appraisal of passive permeability and drug-likeness, whereas pkCSM refined the profile with transporter interactions, total clearance, and CYP-related liabilities. Finally, ADMETlab 3 supplied additional predicted physicochemical and disposition-related parameters including boiling and melting points, as well as predicted total plasma clearance (CL_plasma_) and terminal half-life (t½) thereby completing a physiology-oriented snapshot of the compounds’ anticipated in-vivo behaviour.

Because these platforms use distinct training sets, algorithms, and endpoint definitions, their outputs were not averaged or treated as interchangeable. Comparisons were performed endpoint by endpoint: concordant directional predictions were used for qualitative interpretation, platform-specific numerical values were retained under their original definitions, and discordances were reported as model uncertainty rather than resolved by selecting the most favorable output.

### 2.2 Prediction of the toxicity analysis (Pro Tox III)

Toxicity was predicted with the ProTox‑III web server in accordance with current best‑practice guidelines [24]. The ChemDraw‑derived, validated SMILES strings were submitted to the platform, which combines large toxicological databases with machine‑learning and Bayesian statistics to screen for structural alerts. For each molecule, ProTox‑III reports a spectrum of toxicological end‑points most prominently the predicted acute LD₅₀ and the associated GHS hazard class thereby highlighting potential in‑vivo liabilities. This rapid, high‑resolution assessment supports the rational triage of candidates for subsequent laboratory testing and accelerates data‑driven decisions in both drug‑discovery and environmental‑safety programmes.

### 2.3 PyRx: Preparation, configuration, and validation of the docking protocol

Molecular docking was performed with PyRx [25], which integrates AutoDock Vina [26] and the AutoDock Tools (ADT v1.5.7) interface. The M^pro^ crystal structure was prepared by removing crystallographic waters, adding polar hydrogens, and assigning Gasteiger charges; any residual issues were corrected in Discovery Studio, which was also used after docking to visualise poses and analyse ligand-protein contacts. Ligands were drawn and conformationally optimised in ChemDraw, minimised, and converted to PDBQT format in ADT to ensure proper atom types and charge states. Docking calculations employed a search grid encompassing the catalytic cleft, with Vina’s exhaustiveness set to 8 and all other parameters at their default values.

Docking validation comprised both self-redocking and multi-template cross-docking. The co-crystallized 9C8Q ligand A1AU4 was redocked into the same receptor and grid, reproducing its crystallographic pose with an RMSD of 0.30 Å. For cross-docking, M^pro^ structures 6M2N, 6W63, and 7VU6 were superposed onto chain A of 9C8Q using Cα atoms; the refined receptor RMSDs were 0.425, 0.305, and 0.510 Å, respectively (S1 Fig). The corresponding co-crystallized ligands baicalein, X77, and ensitrelvir were carried into the 9C8Q coordinate frame by the receptor transformation and then independently docked into the unchanged 9C8Q grid. Ligand heavy-atom RMSDs were calculated against the aligned experimental poses without additional ligand-to-ligand fitting; complete results are reported in S1 Table. The neutral 1H-tetrazole tautomer depicted in Scheme 1 was used consistently for docking. Because 5-substituted tetrazoles are ionizable carboxylic-acid bioisosteres, this computational choice should not be interpreted as establishing the dominant microspecies under physiological conditions. Neither the alternative neutral 2H tautomer nor the deprotonated tetrazolate state was evaluated; consequently, the docking rankings are conditional on the selected neutral 1H state and protonation-state sensitivity remains unresolved.

### 2.4 Implementation of molecular dynamics simulations using GROMACS

The entire simulation workflow was executed in GROMACS 2021.3 [27]. Protein topology and coordinates were prepared with gmx pdb2gmx using the AMBER99SB-ILDN force field, including the addition of missing hydrogen atoms and assignment of the corresponding protonation states. Ligands were parameterized independently to generate the required validated.itp and.gro files before assembly of each protein–ligand complex. Each complex was placed at the center of a cubic periodic simulation box with a minimum solute-to-box-wall distance of 1.0 nm, solvated explicitly with TIP3P water molecules, and electrically neutralized by the addition of appropriate counterions without introducing excess salt.

Prior to equilibration, each solvated system was subjected to energy minimization using the steepest-descent algorithm for a maximum of 50,000 steps, with convergence defined by a maximum force below 1,000 kJ·mol ⁻ ¹·nm ⁻ ¹. The minimized systems were subsequently equilibrated in two consecutive stages: first under the NVT ensemble at 310 K, followed by NPT equilibration at 1 bar. Temperature was controlled using the V-rescale thermostat with a coupling constant of τ_t_ = 0.1 ps, independently applied to two coupling groups, Protein_LIG and Water_and_ions, both maintained at 310 K. During NPT equilibration, pressure was regulated isotropically at 1 bar using the Berendsen barostat.

Production MD simulations were then carried out for 100 ns using the leap-frog integrator with an integration time step of 2 fs. A Verlet neighbor-search scheme was employed, with the neighbor list updated every 20 steps (nstlist = 20) and a neighbor-list cutoff of rlist = 1.2 nm. Short-range Lennard–Jones interactions were treated using a force-switch scheme operating between 1.0 and 1.2 nm (rvdw-switch = 1.0 nm; rvdw = 1.2 nm). Long-range electrostatic interactions were calculated using the particle-mesh Ewald (PME) method with rcoulomb = 1.2 nm, a fourth-order interpolation scheme, and a Fourier grid spacing of 0.16 nm. Bonds involving hydrogen atoms were constrained using the LINCS algorithm with constraints = h-bonds, lincs_iter = 1, and lincs_order = 4.

During the production stage, pressure was maintained isotropically at 1 bar using the Parrinello–Rahman barostat, with a pressure-coupling constant of τₚ = 2.0 ps and an isothermal compressibility of 4.5 × 10^−5^ bar ⁻ ¹. Temperature remained controlled at 310 K using the V-rescale thermostat under the same coupling-group definition applied during equilibration. Under these conditions, a 100 ns production trajectory was generated for each protein–ligand system, with coordinates and velocities recorded at regular intervals for subsequent assessment of structural stability, conformational dynamics, and protein–ligand interaction patterns.

All production MD trajectories were generated in explicit TIP3P solvent. The implicit aqueous model was used only for isolated-ligand DFT calculations and for generalized-Born MM/GBSA post-processing. Explicit water molecules could exchange with the active site during MD; however, crystallographic-water occupancy and water-bridge lifetimes were not quantified. Consequently, the present analysis does not assign specific bridge-forming waters as determinants of ligand affinity.

### 2.5 MM/GBSA calculation

Binding free energies (MM/GBSA) were computed with AmberTools 23 (*MMPBSA.py*, parallel mode) from 100 frames extracted every 0.4 ns over the 60–100 ns segment of each GROMACS trajectory. We used the HCT Generalized-Born model (igb = 5) with dielectric constants ε_in_ = 1.0 and ε_out_ = 80.0 and a 0.15 M salt concentration; nonpolar solvation was estimated from the solvent-accessible surface area. Per-residue decomposition (idecomp = 1) was enabled to partition van der Waals, electrostatic, polar, and nonpolar contributions for each residue, and temporary files were removed after execution (keep_files = 0).

### 2.6 DFT study

All quantum-chemical calculations were carried out using ORCA 5.0.4 in an implicit aqueous medium, applying the CPCM (water) solvation model to consistently mimic solution-phase conditions for all investigated ligands and the reference inhibitor. All structures were treated as neutral, closed-shell singlets (q = 0, multiplicity = 1). The neutral 1H-tetrazole input state used for docking was retained to keep the molecular definition consistent across computational layers. This standardization choice does not establish the dominant solution microspecies. Geometry optimizations were performed at the B3LYP-D3(BJ)/def2-SVP level using the RIJCOSX approximation with the def2/J auxiliary basis set, TightSCF convergence criteria, and an adequately fine numerical integration grid. Harmonic frequency calculations were subsequently conducted to confirm that the optimized geometries correspond to true minima on the potential energy surface (no imaginary frequencies). Frontier orbital energies (HOMO and LUMO) were extracted from the converged wavefunctions and used to compute global electronic/reactivity descriptors. Post-processing of ORCA outputs and wavefunctions (Molden format) was performed with Multiwfn 3.8 to ensure consistent descriptor extraction across the full compound set. Finally, HOMO/LUMO isosurfaces were visualized in Avogadro 1.2.0 using identical rendering and isovalue settings to allow direct cross-comparison [28,29].

## 3. Results and discussion

### 3.1 ADME results

#### 3.1.1 Comparative analysis of ADME profiles and drug‑likeness using SwissADME radar plots.

SwissADME radar analysis (Fig 1) shows that the ADME behaviour of the phenyl‑tetrazole chromone series is governed mainly by the tetrazole pharmacophore and the degree of chromone hydroxylation. The parent analogue M1, which lacks hydroxyl groups, combines a hydrophobic chromone scaffold and a phenyl ring with the moderately polar tetrazole, giving high lipophilicity and only modest polarity features that favour passive membrane diffusion yet predict limited aqueous solubility. Addition of a single hydroxyl at C‑3 (M2) or C‑5 (M3) increases the topological polar surface area and hydrogen‑bond‑donor count, markedly boosting predicted solubility while retaining a log P within the optimal window for membrane permeation, thus preserving the physicochemical balance required for oral bioavailability [30]. Double hydroxylation in M4 produces the most symmetrical radar profile: polarity rises enough to confer the best solubility estimate in the series, but lipophilicity remains adequate for trans‑membrane uptake, because the extra hydroxyl enhances hydrogen bonding with aqueous media without tipping the molecule into an excessively polar regime. By contrast, ritonavir presents a radically different pattern: its high molecular mass, pronounced conformational flexibility, strong lipophilicity and substantial polarity collectively impose severe pharmacokinetic liabilities poor water solubility, inefficient gastrointestinal absorption and a reliance on pharmacokinetic boosters stemming from a densely functionalised scaffold rich in amides, carbamates and heteroaromatic rings that foster non‑specific interactions and metabolic load. Taken together, the phenyl‑tetrazole chromone derivatives display a far more drug‑like balance of lipophilicity and polarity than ritonavir, underscoring their potential as orally bioavailable antiviral leads.

**Fig 1:**
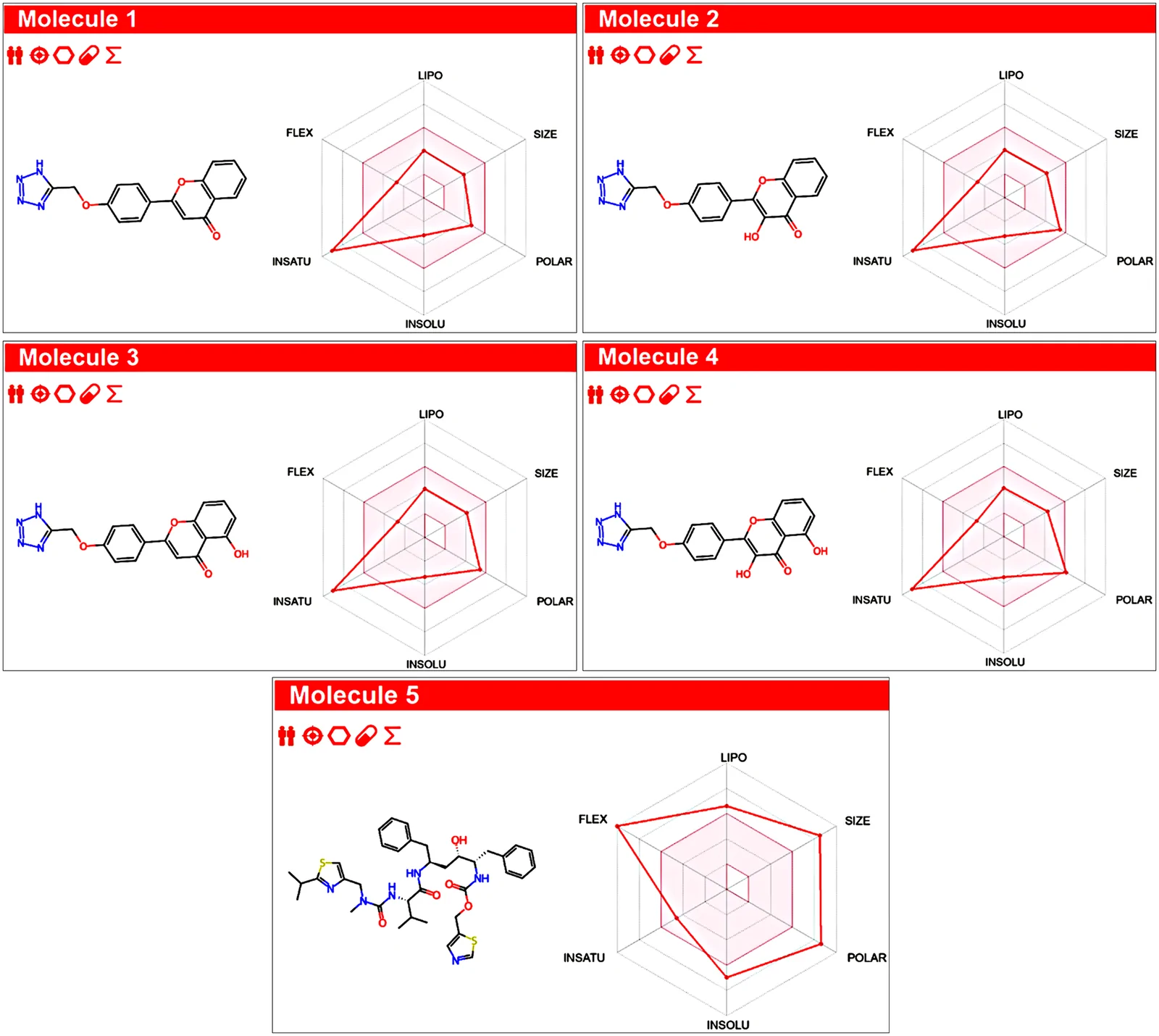
Comparative ADME radar profiles of the phenyl‑tetrazole‑substituted chromone derivatives (M1-M4) and ritonavir (M5) generated with SwissADME.

#### 3.1.2 ADME profiles of the investigated compounds assessed with SwissADME and the “Boiled Egg” model.

A detailed SwissADME assessment (Table 1) shows that the phenyl‑tetrazole chromone derivatives M1-M4 possess far more drug‑like pharmacokinetic profiles than ritonavir. Their molecular masses range from 320.3 to 352.3 g mol ⁻ ¹ less than half that of ritonavir (720.94 g mol ⁻ ¹) a size window that favours passive diffusion through intestinal membranes. Each analogue carries six to eight hydrogen‑bond acceptors and one to three donors, values that remain within oral‑drug guidelines and contrast with ritonavir’s higher polarity load (seven acceptors, four donors). Structural rigidity is also advantageous: the four rotatable bonds found in all four derivatives are a fraction of ritonavir’s 22, limiting entropic penalties and reducing the likelihood of non‑specific binding [31,32].

**Table 1:** Physicochemical, pharmacokinetic, and drug‑likeness properties of the investigated compounds compared with ritonavir.

Molecules	1	2	3	4	Ritonavir
Molecular WEIGHT(g/mol)	320.3	336.3	336.3	352.3	720.94
H-bond acceptors	6	7	7	8	7
H-bond donors	1	2	2	3	4
Rotatable bonds	4	4	4	4	22
TPSA (Å²)	93.9	114.13	114.13	134.36	202.26
CLogP	2.37	1.98	2.24	1.78	5.19
Lipinski: violations	0	0	0	0	2
Veber: violations	0	0	0	0	2
Bioavailability Score	0.56	0.56	0.56	0.56	0.17

Topological polar surface area increases progressively from 93.9 to 134.4 Å² as hydroxyl groups are introduced across the series, yet remains below the empirical 140 Å² ceiling for good absorption; ritonavir’s TPSA of 202.3 Å², by comparison, lies well outside this range and contributes to its poor oral uptake. Calculated lipophilicity (cLogP 1.78–2.37) places M1-M4 in the optimal corridor for simultaneous membrane permeation and aqueous solubility, whereas ritonavir’s cLogP 5.19 predicts low solubility and erratic absorption.

Crucially, all four chromone derivatives satisfy Lipinski and Veber criteria with no violations, and each attains a SwissADME bioavailability score of 0.55. Ritonavir breaches both rules twice and posts a considerably lower score (0.17), reflecting its well‑known need for pharmacokinetic boosting. Taken together, the derivatives’ favourable balance of size, polarity, rigidity and lipophilicity supports their candidacy as orally available antiviral leads, whereas ritonavir’s excessive molecular bulk, high TPSA and extreme lipophilicity underscore its pharmacokinetic liabilities.

The phenyl‑tetrazole chromone series displays ADME profiles that are markedly more drug‑like than those of ritonavir, translating into superior oral pharmacokinetic prospects.

Boiled‑Egg analysis (Fig 2) further illustrates how incremental hydroxylation modulates human intestinal absorption (HIA) and blood-brain‑barrier (BBB) permeability [33]. The parent molecule M1, bearing no hydroxyl groups, lies near the HIA perimeter yet still outside the BBB zone, a placement consistent with its balanced lipophilicity (WLogP ≈ 2.4) and moderate polarity (TPSA ≈ 94 Å²). Single hydroxylation at C‑3 (M2) or C‑5 (M3) shifts the centroid deeper into the optimal HIA region while moving it farther from the BBB boundary; the added polarity (TPSA ≈ 114 Å²) enhances aqueous solubility and gut uptake but reduces CNS penetration, making these analogues attractive for peripheral indications. Double hydroxylation in M4 pushes the marker to the very edge of the BBB‑excluded field at a TPSA of ≈ 134 Å², virtually eliminating central exposure while preserving excellent intestinal permeability an advantageous profile for treatments where CNS activity is undesirable. Ritonavir, by contrast, would plot well outside the egg entirely: its high polarity (TPSA ≈ 202 Å²) and extreme lipophilicity (WLogP > 5) predict poor oral absorption and negligible BBB passage, a consequence of its bulky, multifunctional scaffold.

**Fig 2:**
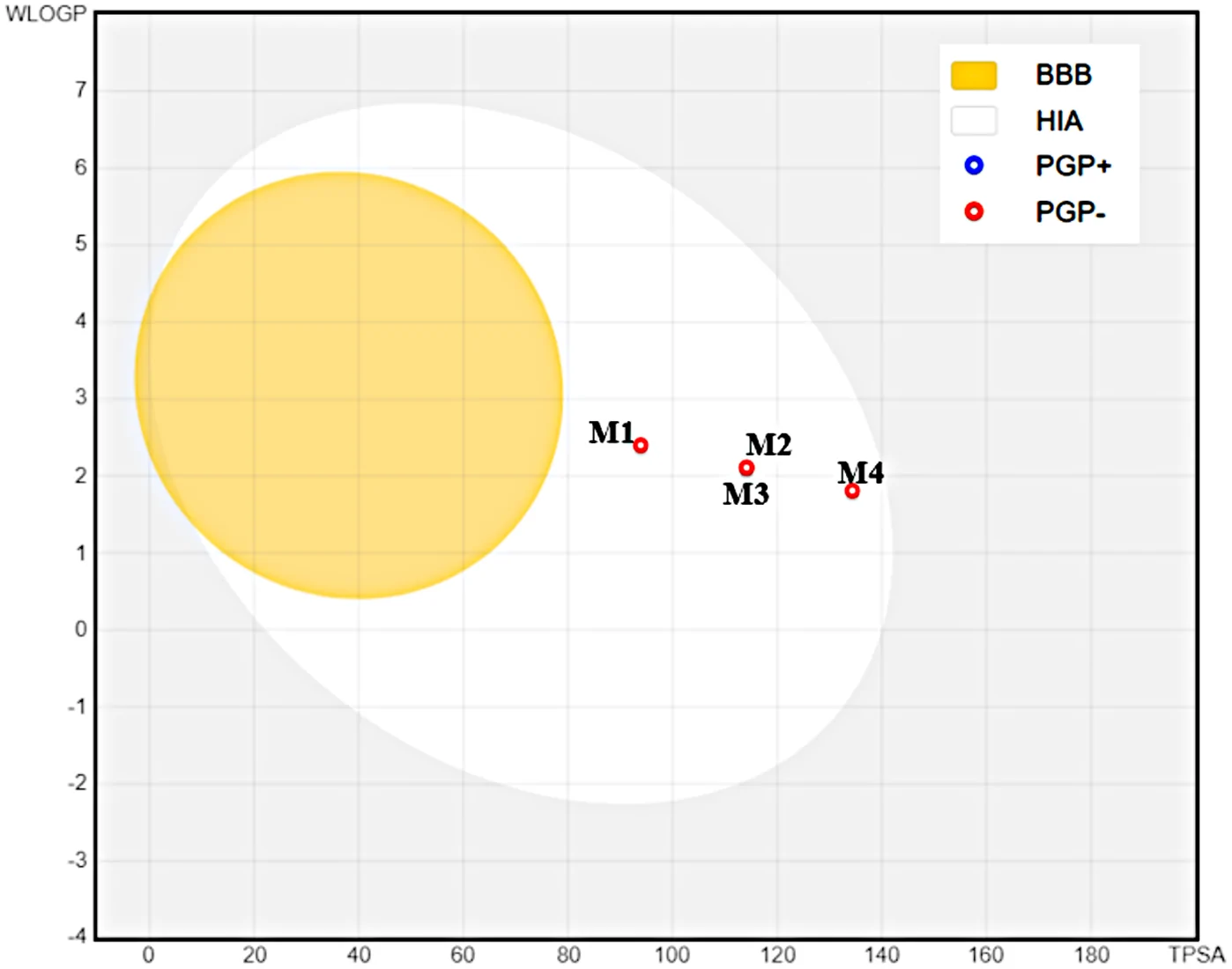
Boiled‑Egg plot assessing intestinal absorption and blood-brain‑barrier permeability of the phenyl‑tetrazole chromone derivatives (M1-M4).

Collectively, the Boiled‑Egg positions of M1-M4 confirm that these phenyl‑tetrazole chromones combine efficient intestinal absorption with tunable CNS liability, offering therapeutic flexibility that ritonavir lacks.

#### 3.1.3 Pharmacokinetic and metabolic consequences of incremental polarity modulation insights from pkCSM.

According to pkCSM in-silico predictions, the data in Table 2 reveal that the stepwise increase in polarity across the M1 → M4 series tightly dictates their pharmacokinetic behaviour: as tPSA rises from ≈ 93 Å² (M1) to ≈ 134 Å² (M4), predicted human intestinal absorption falls from 82% to 71%, exemplifying the classical inverse relationship between polar surface area and passive permeability. Introducing a single hydroxyl at C-3 (M2) or C-5 (M3) produces only a modest drop (< 3%), because one OH group remains compatible with the enterocyte’s lipophilic corridor, whereas di-hydroxylation in M4 lowers lipophilicity below the optimal window and hampers transcellular diffusion. Ritonavir, far bulkier (720 g mol ⁻ ¹) and more polar (tPSA ≈ 202 Å²), achieves only 62% absorption, consistent with its need for solubilising excipients in vivo.

**Table 2:** pkCSM-predicted ADMET parameters for chromone-tetrazole derivatives (M1-M4) versus ritonavir.

Molecules	M1	M2	M3	M4	Ritonavir
Intestinal absorption (human) %	82	79	76	71	62
BBB permeability	−1.28	−1.5	−1.5	−1.8	−2.082
CNS permeability	−2.82	−3.86	−3.71	−4.03	−3.36
CYP2D6 inhibitor	No	No	No	No	No
CYP3A4 inhibitor	Yes	No	No	No	Yes

SwissADME and pkCSM were directionally concordant in predicting low BBB access and a progressive permeability penalty with increasing hydroxylation, but their absorption outputs are not numerically interchangeable: the SwissADME BOILED-Egg is a categorical physicochemical classifier, whereas pkCSM reports a model-specific percentage estimate. ADMETlab 3.0 contributed different endpoints, including predicted clearance, half-life, and thermal properties, rather than an independent measurement of the same absorption quantity. Accordingly, the interpretation was based on cross-platform qualitative concordance, and isolated numerical differences were retained as prediction uncertainty.

pkCSM’s blood-brain barrier indices (logBB) and parenchymal permeability scores (logPS) are strongly negative (<−1 and <−2.5, respectively) for all derivatives, indicating negligible CNS penetration; the ranking M1 > M2 ≈ M3 > M4 mirrors the tPSA gradient, while ritonavir suffers an additional penalty from its 22 rotatable bonds, yielding logBB = −2.08. Such limited brain access immediately reduces the risk of neurological side effects, a desirable feature for systemic antiviral or antibacterial agents [34].

pkCSM classified M1 and ritonavir as CYP3A4 inhibitors, whereas M2-M4 were classified as non-inhibitors. Because this output is a ligand-based binary prediction, it cannot distinguish a purely steric origin from electronic, hydration, desolvation, or lipophilicity effects. Hydroxylation changes several of these properties simultaneously; therefore, the classification shift should be interpreted as a structure-dependent prediction rather than evidence that hydroxyl groups directly disrupt a defined CYP3A4 binding mode. The universal lack of predicted CYP2D6 inhibition is likewise reported as a model output, without assigning a specific residue-level mechanism.

To examine the pkCSM classification structurally, M1-M4 were docked into the validated ritonavir-bound CYP3A4 structure (PDB 3NXU; S1 Method). All four compounds occupied the heme-adjacent active-site cavity, with AutoDock Vina scores of −9.5, −9.0, −9.0, and −8.8 kcal mol-1 for M1-M4, respectively (S2 Fig). M1 retained the most favorable score, whereas the hydroxylated analogues remained sterically accommodated but showed modestly less favorable values. The 0.5–0.7 kcal mol^-1^ separation is small and does not support a simple steric-exclusion mechanism. Accordingly, the pkCSM shift from M1 to M2-M4 is best interpreted as a model-specific response to the combined changes in polarity, lipophilicity, desolvation, and binding orientation introduced by hydroxylation, rather than proof of either a purely electronic effect or purely steric hindrance. The supplementary docking demonstrates active-site compatibility only; it does not establish CYP3A4 inhibition, metabolic turnover, heme coordination, or pharmacokinetic boosting. These questions require recombinant-enzyme inhibition and microsomal-stability experiments.

#### 3.1.4 Structure-driven correlation between thermal properties and pharmacokinetic behaviour.

Fig 3 vividly illustrates how subtle structural tuning dictated by polarity, planarity, and steric bulk modulates both thermal stability and pharmacokinetic fate across the chromone-tetrazole series (M1-M4) versus ritonavir.

**Fig 3:**
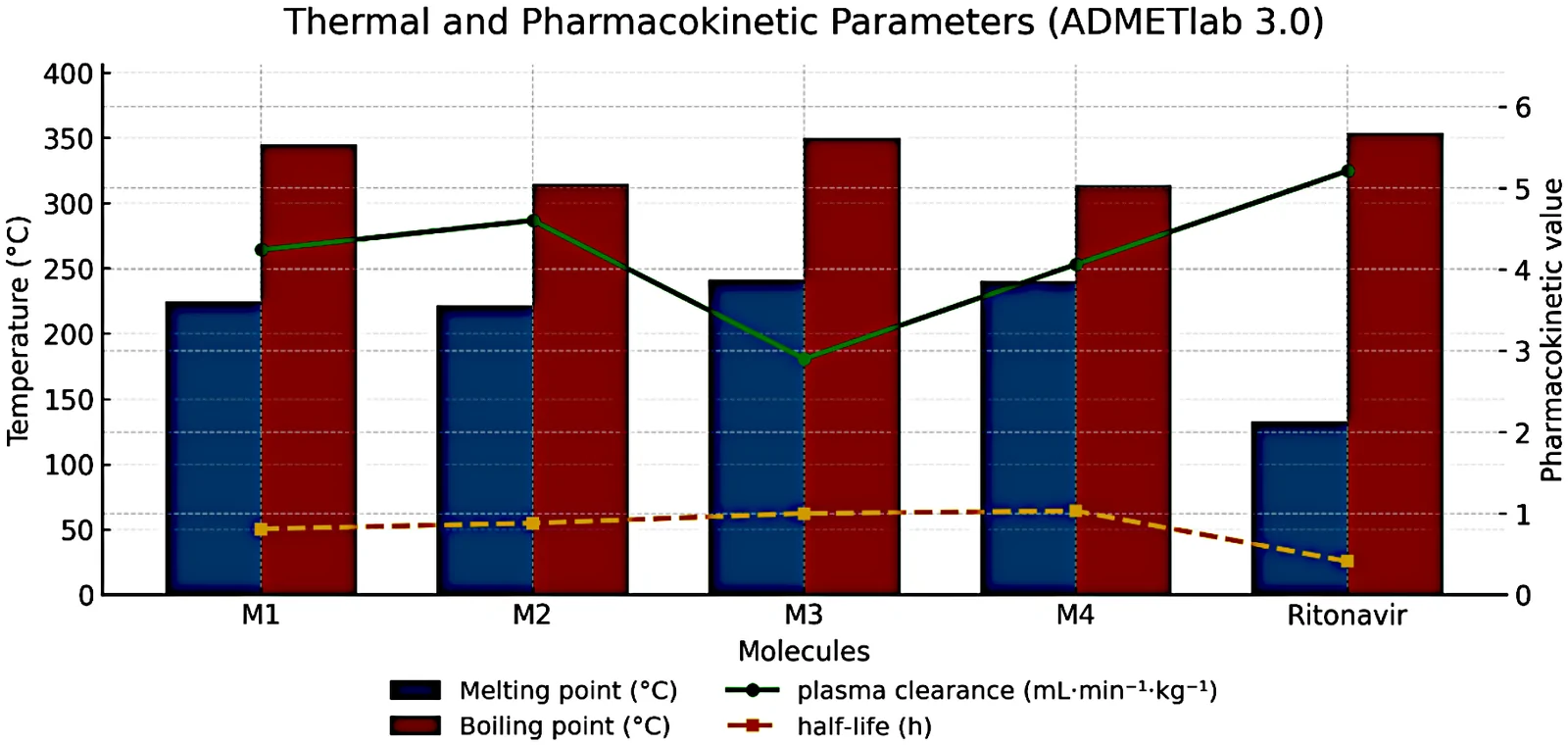
Thermal parameters (melting and boiling points) plotted against predicted pharmacokinetic indices (plasma clearance and terminal half-life) for chromone-tetrazole derivatives M1-M4 compared with ritonavir.

For M1, the absence of hydroxyl groups preserves an uninterrupted, coplanar chromone-phenyl array that stacks efficiently in the crystal lattice, giving a high melting point (~224 °C) and an even higher boiling point (~344 °C) [35]. With a log P near 2.4 and just one rotatable O-CH_2_ bond, hepatic uptake via OATP is rapid; the model therefore predicts a moderate plasma clearance (~4.2 mL· min ⁻ ¹· kg ⁻ ¹) and a short terminal half-life (t½ ≈ 0.8 h). Introducing a C-3 phenolic OH in M2 disrupts π-stacking through an intramolecular H-bond, loosening the lattice (mp 221 °C; bp 314 °C). The exposed phenol is an excellent UGT substrate, which drives the highest clearance in the series (~4.6) and only a slight half-life extension (≈ 0.9 h). Conversely, the C-5 OH in M3 forms an intermolecular H-bond without disturbing planarity, thus reinforcing crystal packing (mp 241 °C; bp 349 °C). Partial shielding of the tetrazole reduces metabolic exposure, lowering clearance (~2.9) and conferring the longest t½ (~1.0 h). The di-hydroxylated M4 (C-3/C-5) resurrects competing O-H···O contacts: the melting point remains high (~240 °C) but the boiling point drops (bp 313 °C) as hydrogen-bonded clusters vaporise more readily. Two phenolic sites accelerate glucuronidation, so clearance rises (~4.1) and t½ increases only marginally (~1.0 h).

Ritonavir represents the opposite extreme. Its flexible, peptidomimetic scaffold riddled with rotatable bonds and carbamate/urea hinges prevents orderly stacking, yielding a very low mp (~132 °C). In the vapour phase, numerous heteroatoms form extensive intermolecular H-bonds, inflating the bp (~353 °C). High basicity and conformational freedom expose the molecule to intense CYP3A4 turnover, explaining the greatest predicted clearance (~5.2) and the shortest t½ (~0.4 h) [36].

Collectively, Fig 3 demonstrates that the interplay of aromatic planarity, hydrogen-bond density, and conformational rigidity simultaneously governs crystalline robustness and systemic elimination rates: a single hydroxyl can invert the mp/bp hierarchy and shift the clearance-half-life balance, while ritonavir’s bulky peptidic architecture shows the structural limits of this structure-function relationship.

### 3.2 Structure-toxicity relationship of the investigated compounds compared with ritonavir

ProTox‑III acute‑toxicity predictions highlight the structural determinants of safety in the phenyl‑tetrazole chromone series (M1-M4). M1 and M3, which carry zero and one hydroxyl group, respectively, fall into toxicity class 5 with projected LD₅₀ values of 4 000 mg kg ⁻ ¹ indicative of very low acute risk (Fig 4) [37]. These values are model-based screening estimates rather than experimental hazard measurements. The approximately 69% average similarity and 68% model precision support cautious within-series interpretation but not a definitive safety classification.

**Fig 4:**
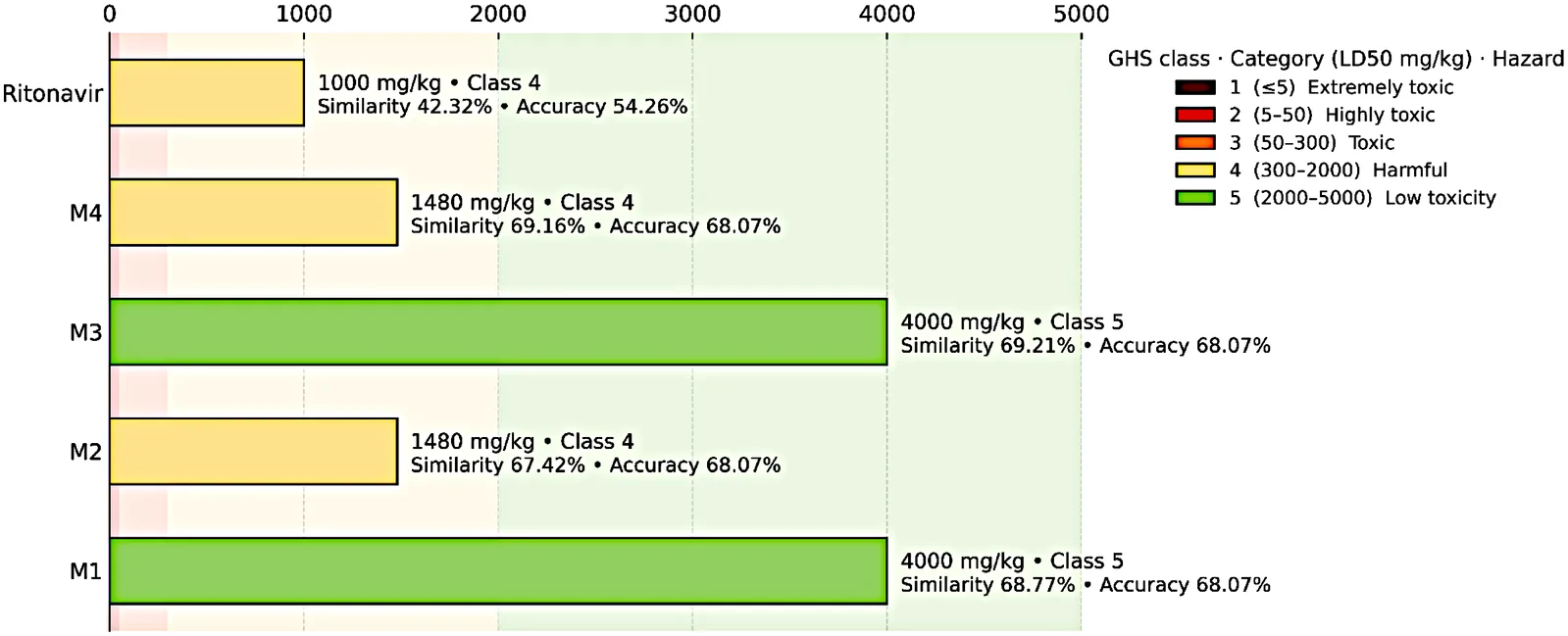
Acute oral toxicity (LD₅₀, rat) with GHS classification for M1-M4 and ritonavir (0-5000 mg/kg scale; annotated with average similarity and prediction accuracy).

Single (M2) or double (M4) hydroxylation shifts the compounds into predicted toxicity class 4, with an estimated LD₅₀ of 1,480 mg kg ⁻ ¹. Although the added hydroxyl groups alter polarity and potential metabolic susceptibility, ProTox-III alone cannot establish quinone formation or a causal structure-toxicity mechanism. The similar confidence metrics (average similarity approximately 68%; precision 68%) support an internal comparison while retaining substantial prediction uncertainty.

Ritonavir has a predicted LD₅₀ of 1,000 mg kg ⁻ ¹ (class 4), but its average similarity of 42% and precision of 54% indicate a weaker applicability-domain match. This low confidence limits ritonavir’s use as a quantitative toxicological benchmark. It does not directly alter the independently generated predictions for M1-M4, but it weakens any conclusion that the hybrids are safer than ritonavir on the basis of predicted LD₅₀ values alone.

Overall, the phenyl-tetrazole chromone derivatives show low-to-moderate predicted acute-toxicity signals within the ProTox-III model. These results are hypothesis-generating, and both the within-series ranking and any comparison with ritonavir require experimental cytotoxicity and toxicological validation.

### 3.3 Molecular docking

To launch the docking investigation, we employed the high‑resolution crystal structure of the SARS‑CoV‑2 main protease (M^pro^, PDB 9C8Q; 1.69 Å) (Fig 5). This homodimeric cysteine protease whose catalytic activity hinges on the Cys145-His41 dyad and the Glu166 gatekeeper is indispensable for viral polyprotein cleavage and lacks close human homologues, making it an ideal target for selective inhibition [4,38–40]. The search grid was centred on the S1/S2 region to include the functional triad His41-His163-Glu166 and the hydrophobic vestibule framed by Cys145. This setup allows each analogue’s ability to form key hydrogen bonds and to anchor its phenyl‑tetrazole moiety within the hydrophobic pocket to be compared within a common, structure-specific docking setup; these contacts remain computational hypotheses pending biochemical validation.

**Fig 5:**
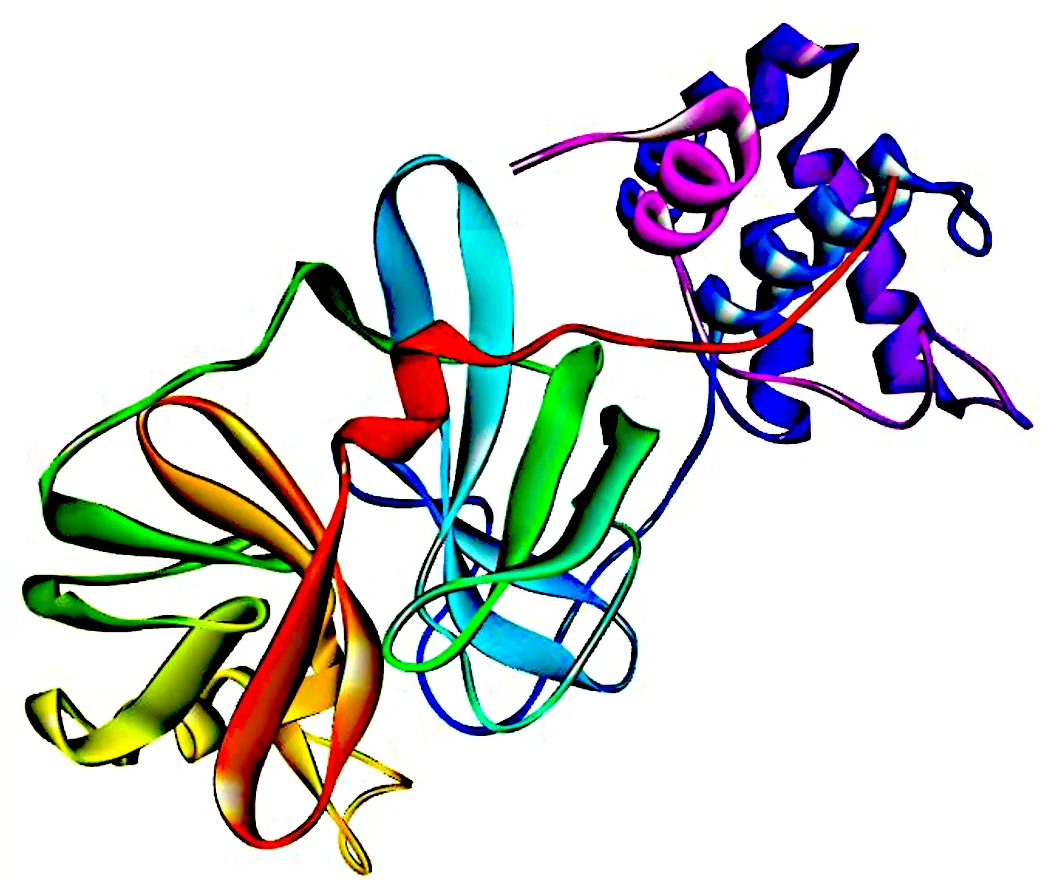
3D representation of the crystal structure of the SARS‑CoV‑2 main protease (PDB 9C8Q).

Table 3 presents a narrow spread of docking energies for the chromone‑phenyl‑tetrazole series (‑7.2 to ‑7.8 kcal mol ⁻ ¹), yet a qualitative analysis of the binding modes reveals marked differences in how each analogue engages the catalytic site. M2 achieves the most favourable (‑7.8 kcal mol ⁻ ¹) by forging a dual hydrogen‑bond clamp with the catalytic His41 (2.91 Å) and Phe140 (3.00 Å); this polar “pincer” locks the S1′ loop and stabilises the S1 hinge, while the phenyl‑tetrazole moiety nests in the S2 hydrophobic vestibule, yielding an optimal enthalpy-entropy balance. M1 (‑7.7 kcal mol ⁻ ¹) adds an additional contact to Glu166 (2.20–3.40 Å), but the resulting, more superficial orientation diminishes hydrophobic burial and thus limits the energy gain [41–43]. M4 (‑7.5 kcal mol ⁻ ¹) benefits from a very short hydrogen bond to His163 (1.99 Å) and a Met165/Glu166 tripod, yet the di‑hydroxylation increases desolvation penalties and attenuates lipophilic contributions. M3, lacking direct anchoring to His41, relies on a Phe140/Glu166 clamp (2.62 Å and 3.21–2.12 Å) and therefore records the least favourable (‑7.2 kcal mol ⁻ ¹). By comparison, the reference ritonavir (‑8.6 kcal mol ⁻ ¹) derives its superior affinity from a contact surface nearly three times larger and two ultra‑short hydrogen bonds to Glu166 (1.92/ 2.47 Å), albeit at the cost of excessive mass, flexibility and an unfavourable ADME profile. Collectively, M2 emerges as the best compromise: it simultaneously targets the catalytic dyad via His41, locks the S1 loop through Phe140, exploits Glu166 to close the catalytic channel, and retains the compactness and rigidity conducive to high oral bioavailability.

**Table 3:** Predicted binding affinities (kcal mol ⁻ ¹) and key hydrogen bonds formed by chromone‑phenyl‑tetrazole derivatives (M1-M4) and ritonavir within the SARS‑CoV‑2 main protease (M^pro^, PDB 9C8Q).

Molecules	Docking Score(Kcal/mol)	Hydrogen bonds	Distance (Å)
**M1**	−7.7	His41, Phe140, Glu166	2.93; 2.67; (2.2;3.40)
**M2**	−7.8	His41, Phe140	2.91; 3.00
**M3**	−7.2	Phe140, Glu166	2.62; (3.21;2.12)
**M4**	−7.5	His163, Met165, Glu166	1.99; 3.36; 3.14
**Ritonavir**	−8.6	Glu166	1.92; 2.47

Overall, the interaction profiles of the four compounds indicate coherent targeting of the M^pro^ active site (His41-Cys145 dyad), with a preferred anchoring in the S1 subpocket (Phe140/Glu166/His163) and hydrophobic reinforcement in S2 (Met49/Met165): for compound 1 (Fig 6), a triad of conventional hydrogen bonds C = O···His41 plus two bonds from the tetrazole to Glu166 and Phe140 locks the pharmacophore in place, while two π-alkyl contacts with Met49, two π-sulfur contacts with Cys44, an amide-π stack with Met165, a π-σ contact with Glu166, and a π-cation interaction with His41 densify the interface; only an unfavorable donor-donor contact with His172 suggests a minor geometric penalty [44–46]. Compound 2 (Fig 7) retains a lean H-bond network (Phe140 and His41) but leans more on hydrophobic/chalcogen contributions: π-alkyl to Met49, π-sulfur to Met49 plus two π-sulfur contacts to Cys44, amide-π with Met165, π-σ with Glu166, and π-cation with His41; around His172, an unfavorable donor-donor contact coexists with a compensatory carbon-hydrogen bond. Compound 3 (Fig 8) reiterates the directing role of the tetrazole via two H-bonds to Phe140 and Glu166 that orient and rigidify S1 anchoring, complemented by a versatile bundle of π-alkyl/π-σ/amide-π contacts (Met49, Met165), π-sulfur (Cys44), and carbon-hydrogen bonding (notably with Glu166), with again an unfavorable donor-donor contact toward His172 to monitor [47,48]. Finally, compound 4 (Fig 9) stands out through an H-bond to His163, a pronounced π-sulfur interaction with the catalytic cysteine Cys145, and a T-shaped π-π contact with His41, a trio that signals optimal catalytic engagement at the core of the dyad, reinforced by a carbon-hydrogen bond with Glu166 and additional hydrophobic contacts; in sum, M1 and M3 derive stability from a tetrazole-driven S1 “lock” supported by S2 (Met49/Met165), M2 emphasizes a stronger chalcogen-mediated lateral cohesion, and M4 exhibits the most inhibition-prone configuration via its dual, direct contacts to Cys145 and His41.

**Fig 6:**
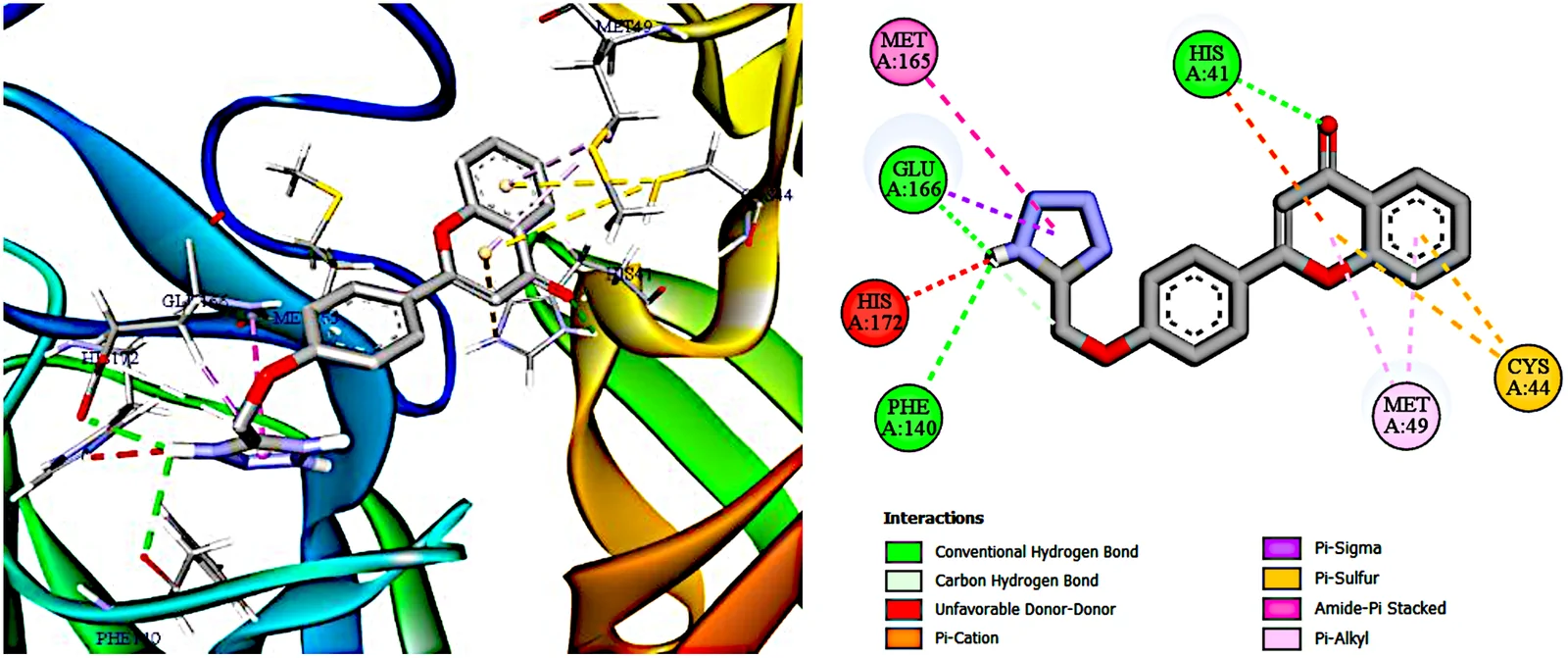
2D/3D binding interactions of compound M1 with the SARS-CoV-2 main protease (M^pro^) active site.

**Fig 7:**
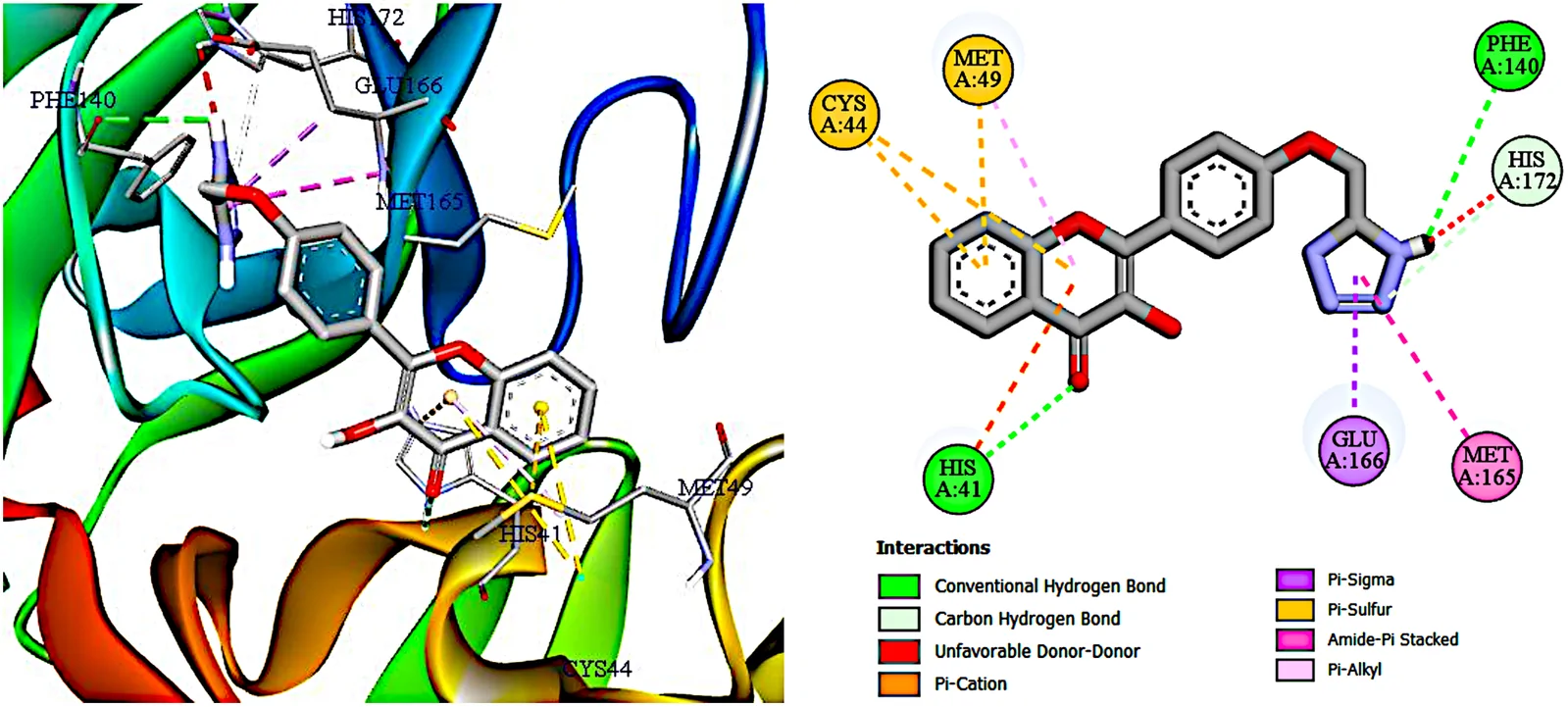
2D/3D binding interactions of compound M2 with the SARS-CoV-2 main protease (M^pro^) active site.

**Fig 8:**
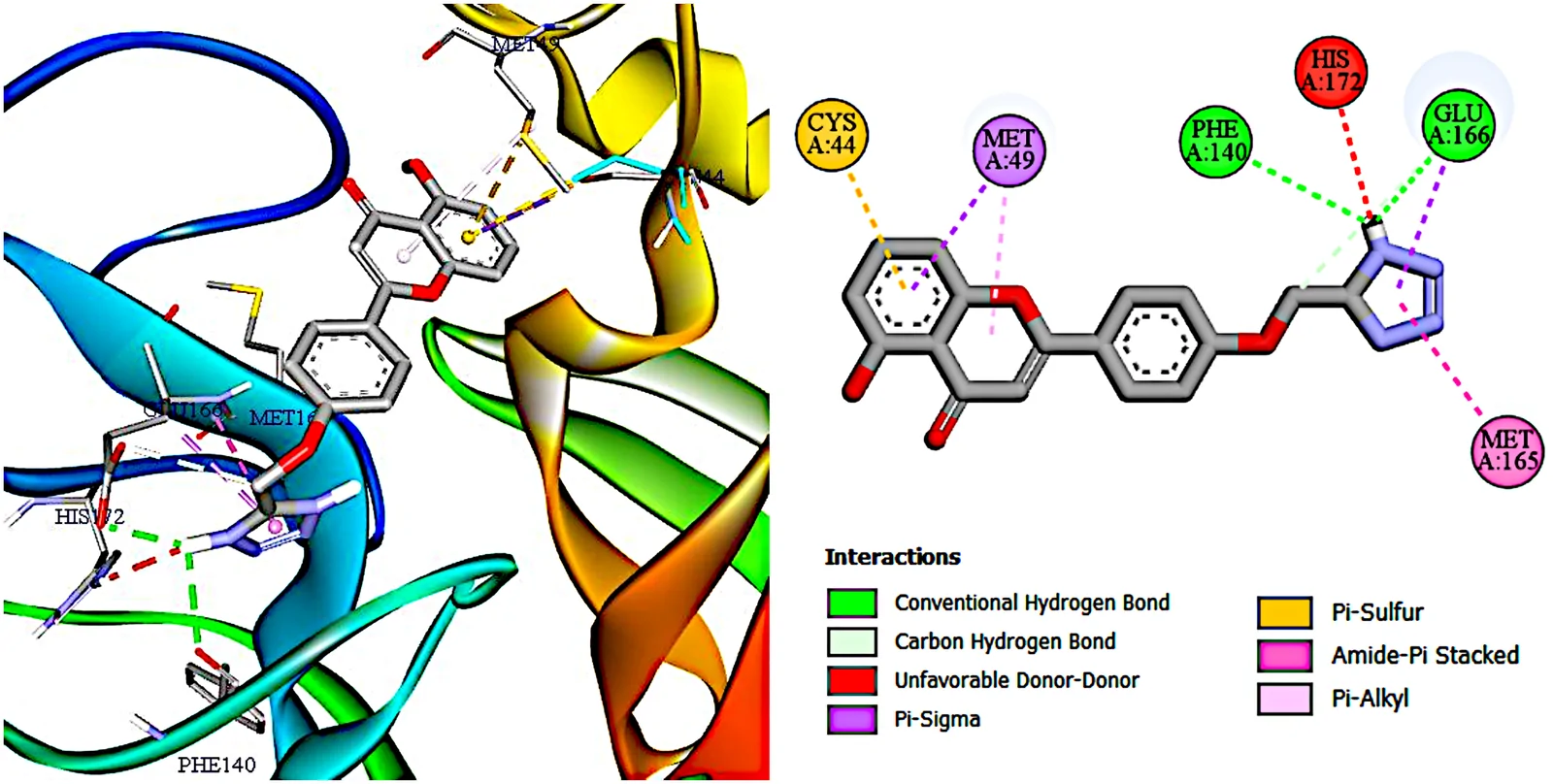
2D/3D binding interactions of compound M3 with the SARS-CoV-2 main protease (M^pro^) active site.

**Fig 9:**
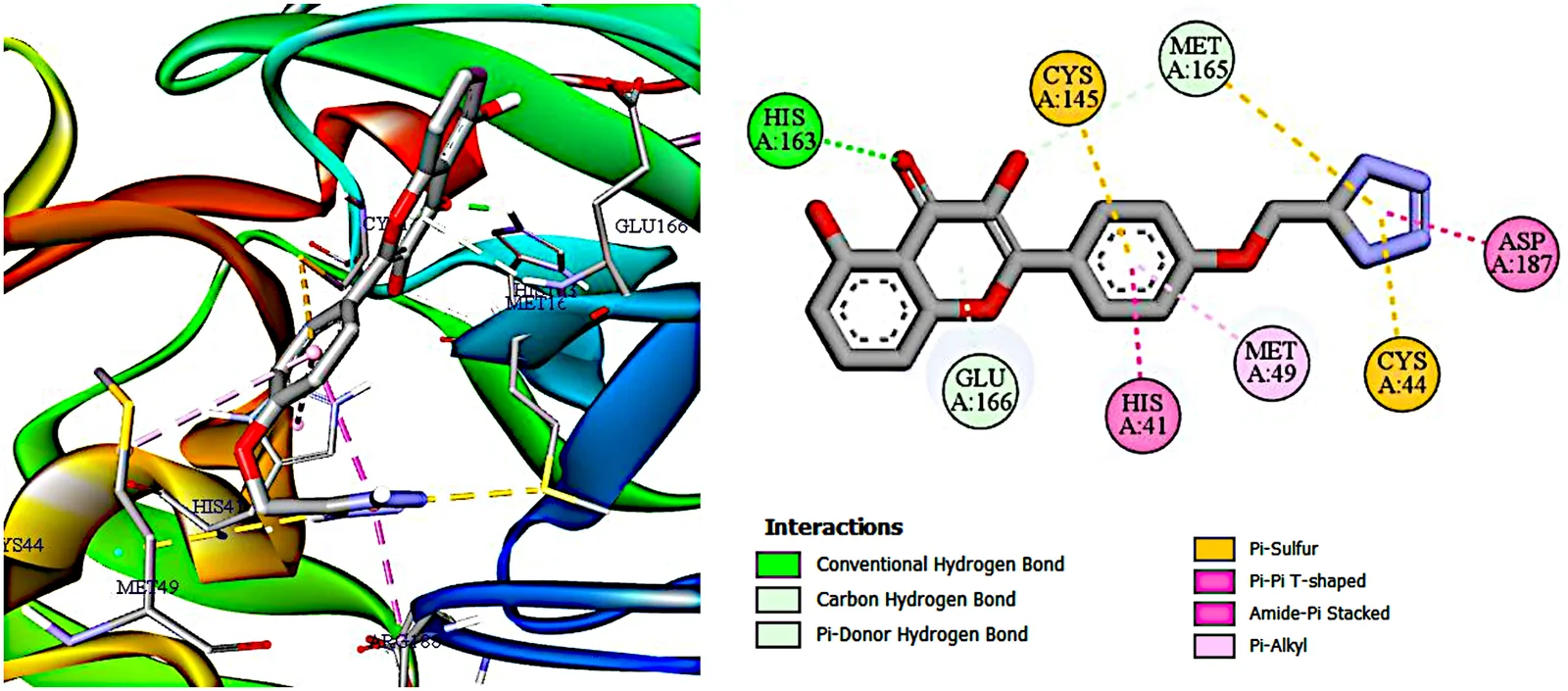
2D/3D binding interactions of compound M4 with the SARS-CoV-2 main protease (M^pro^) active site.

### 3.4 Molecular dynamics simulation

Molecular dynamics (MD) complements docking by testing whether a docked pose remains stable in an explicitly solvated, fully flexible environment; taken together, RMSD (global positional drift), RMSF (per-residue flexibility), radius of gyration Rg (overall compactness), and hydrogen-bond (HB) time profiles provide orthogonal criteria to confirm or challenge docking hits. In practice, a convincing binder shows a low and plateaued ligand RMSD after equilibration, protein RMSF confined to loop/termini without new hotspots around the pocket, an Rg that is steady (no global swelling/collapse), and one to a few persistent HBs that cooperate with hydrophobic packing exactly the type of dynamic evidence needed to validate the four top-scoring docked compounds [49].

In Fig 10 (ligand RMSD), M2-M4 remain tightly bound with sub-nanometric drifts (~0.15–0.35 nm) that quickly converge and stay flat over 100 ns, indicating pose stability and limited reorientation in the pocket; by contrast, M1 displays a large drift that rises toward ~1.0 nm with episodic spikes approaching ~2.0 nm around the mid-trajectory, consistent with pose hopping or partial disengagement and therefore a weaker dynamic endorsement of its docking pose [50].

**Fig 10:**
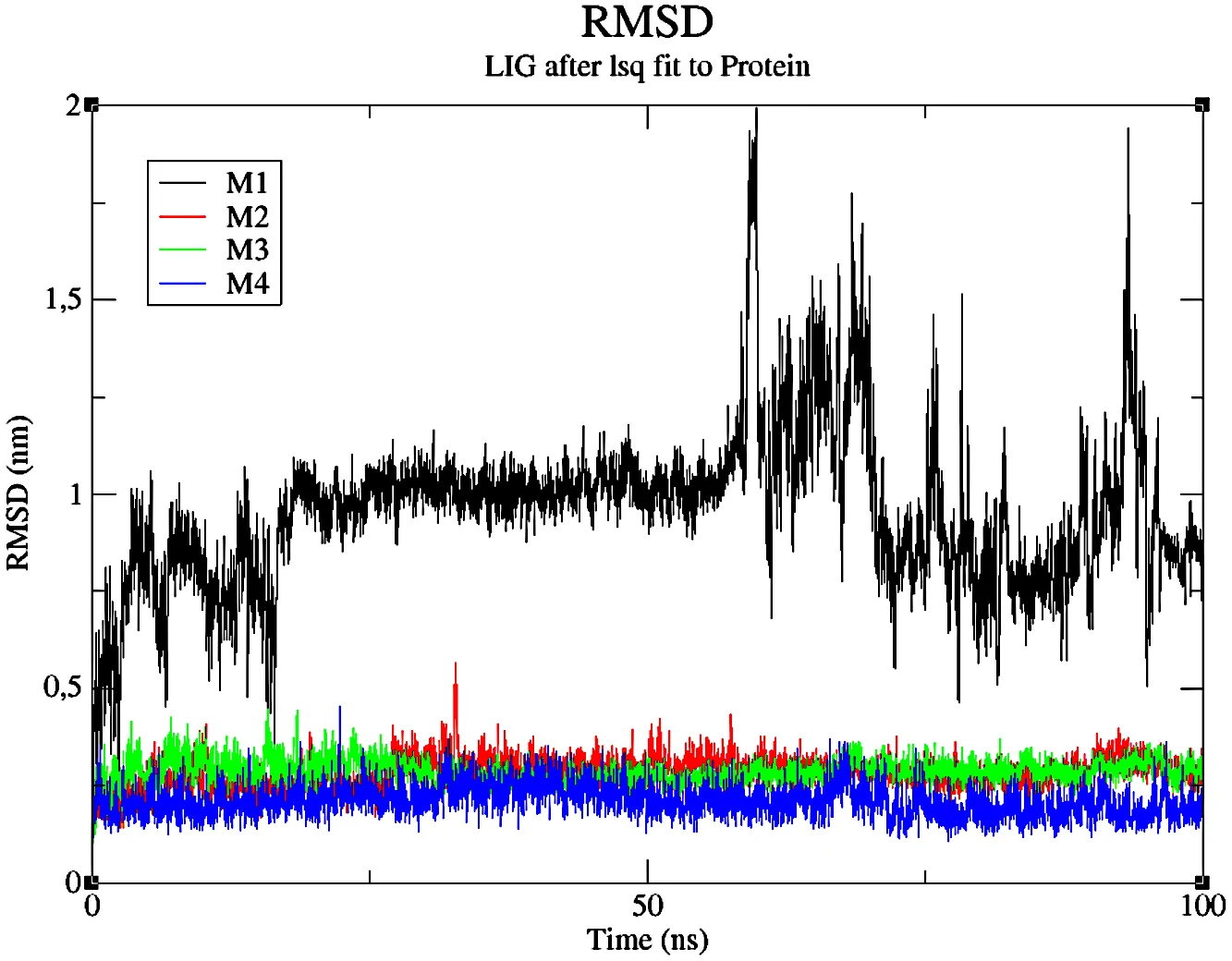
Time evolution of ligand RMSD for M1-M4 bound to the SARS-CoV-2 main protease (M^pro^) over 100 ns.

Fig 11 shows that backbone RMSF profiles for all complexes are low and similarly shaped across the sequence, with the expected peaks at terminal/loop segments and no emergence of new flexibility around the binding site; M4 exhibits slightly higher fluctuations at a few loop regions but still within a restrained range (<0.5 nm). The paired Rg traces indicate preserved global compactness for all systems; P-M3 is the most compact and stable (Rg ~ 2.16–2.20 nm with minimal drift), P-M1 and P-M2 are modestly higher yet steady (~2.20–2.24 nm), while P-M4 shows a gentle increase toward ~2.30 + nm late in the run, suggestive of minor breathing rather than unfolding.

**Fig 11:**
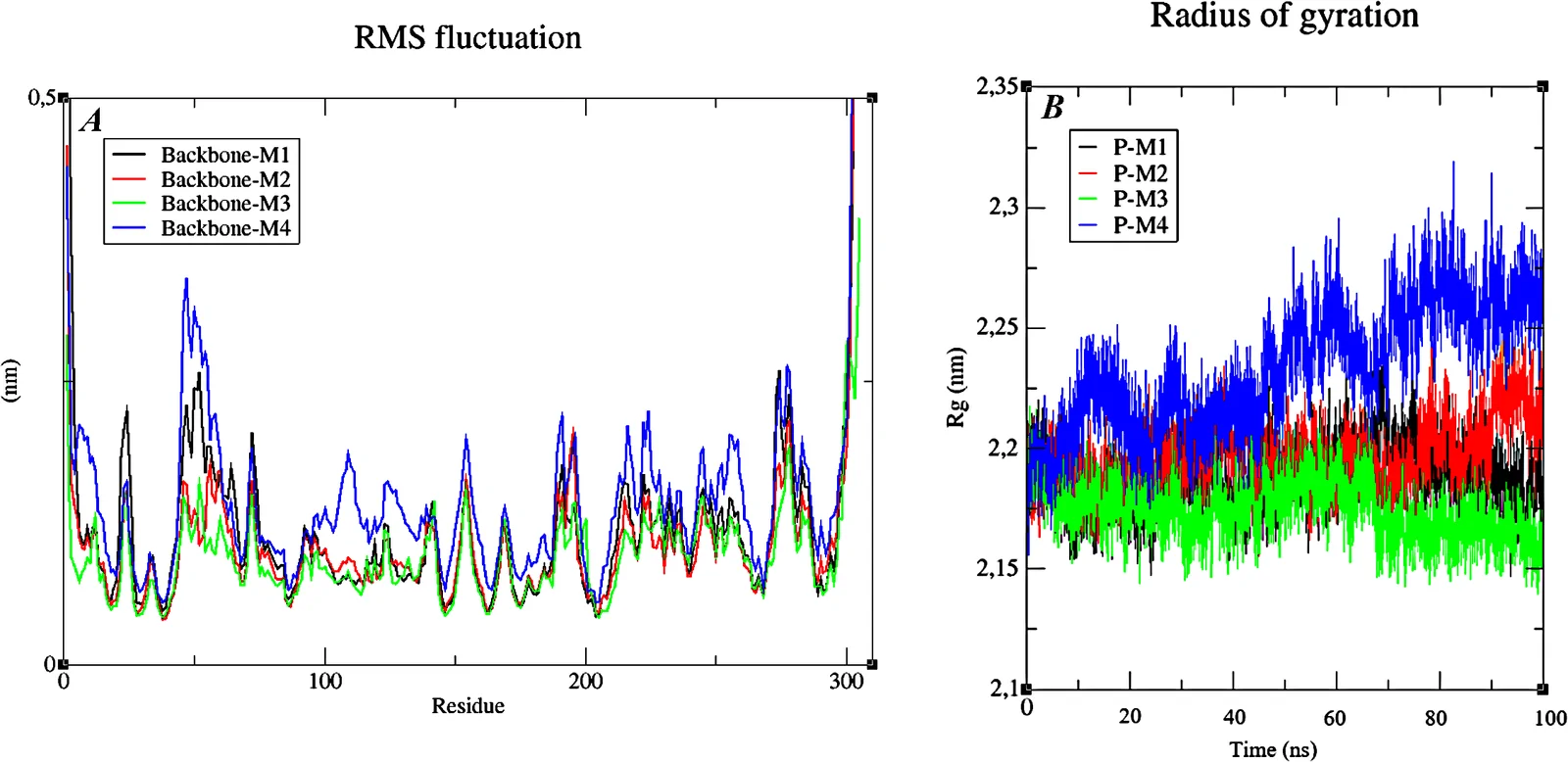
Protein flexibility and compactness during MD for M1-M4 complexes: (A) residue-wise backbone RMSF of M^pro^, (B) radius of gyration (Rg) of M^pro^ over 100 ns.

In Fig 12 (ligand-protein hydrogen bonds), M4 sustains a consistently populated network of ~2–3 HBs across most of the simulation, matching its low RMSD and supporting durable anchoring; M2 and M3 maintain predominantly 1–2 HBs with intermittent spikes, which is compatible with stable but somewhat more hydrophobically driven binding; M1 shows a broad, transient distribution (0 up to ~6–7 HBs around 30–60 ns) that fluctuates markedly, aligning with its high RMSD and indicating pose rearrangements. Altogether, MD corroborates the docking for M2-M4 especially M4, and to a compactness extent M3 while cautioning that M1’s docked pose is not dynamically robust.

**Fig 12:**
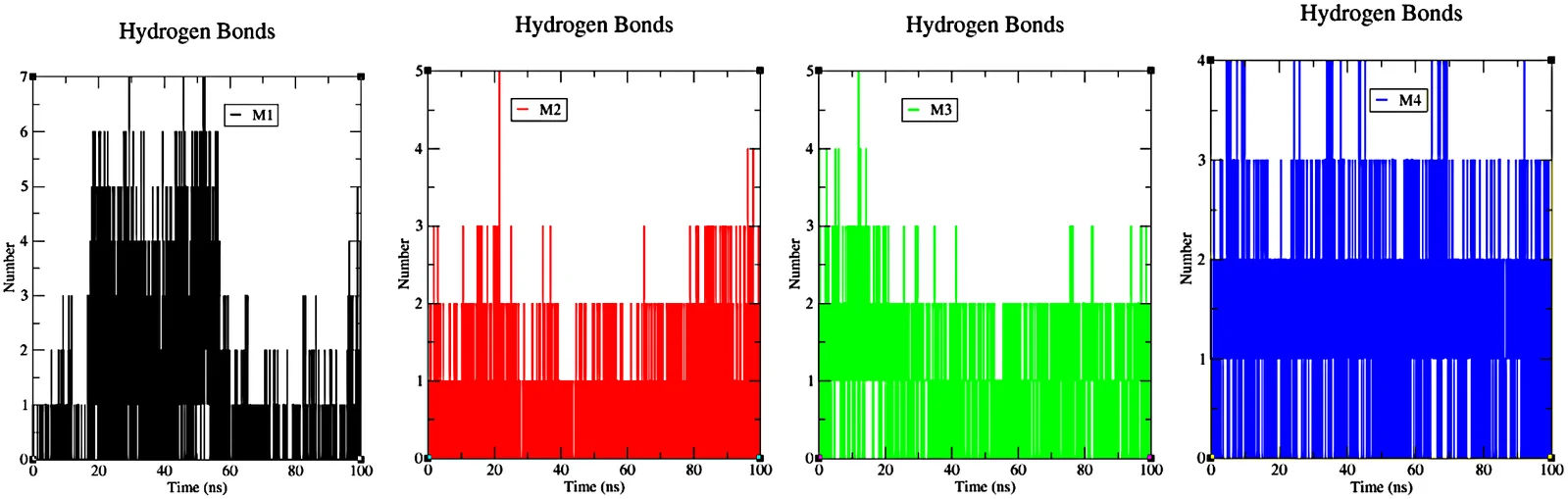
Number of ligand-protein hydrogen bonds versus time for M1-M4 complexes with M^pro^ over 100 ns.

### 3.5 MM/GBSA free-energy analysis

MM/GBSA analysis. Binding free energies (ΔG_bind_, kcal·mol ⁻ ¹) were estimated from 100 equilibrated MD snapshots collected between 60–100 ns. MM/GBSA complements docking and MD by partitioning the interaction into gas-phase terms van der Waals and electrostatics (ΔG_gas_ = ΔE_vdW_ + ΔE_ele_) and solvent response polar GB and non-polar surface contributions (ΔG_solv_ = ΔE_GB_ + ΔE_surf_). As summarized in this Table 4, all three complexes (M2-M4) exhibit negative ΔG_bind_ in water, indicating thermodynamically favorable binding [51].

**Table 4:** MM/GBSA binding free-energy decomposition (kcal·mol ⁻ ¹) for complexes M2-M4.

	Kcal/mol		
Molecules	M2	M3	M4
ΔE_vdW_ (vdW interactions)	−46.48	−37.99	−30.76
ΔE_ele_ (electrostatic)	0.00	−151.51	−92.32
Δ_EGB_ (polar solvation)	12.84	159.38	104.51
ΔE_surf_ (non-polar solvation)	−5.13	−4.81	−3.99
ΔG_gas_ (gas-phase)	−46.48	−189.50	−123.08
ΔG_solv_	7.71	154.57	100.52
ΔG_binding_	−38.77	−34.93	−22.56

$\Delta G_{\text{bind }}=\Delta G_{\text{gas }}+\Delta G_{\text{solv }}=\left(\Delta E_{\text{vdW }}+\Delta E_{\text{elec }}\right)+\left(\Delta E_{\text{GB}}+\Delta E_{\text{surf }}\right)(kcal\cdot mol^{\text{-1}})$ Table 4 shows negative binding free energies for all complexes ΔG_bind_ = −38.8, −34.9, and −22.6 kcal·mol ⁻ ¹ for M2, M3, and M4, respectively ranking M2 > M3 > M4 in predicted affinity. The driving forces clarify this trend: M2 is dominated by favorable dispersion (ΔE_vdW_) with minimal electrostatics (ΔE_ele_ ≈ 0) and a modest desolvation cost (ΔG_solv_ ≈ +7.7 kcal·mol ⁻ ¹), yielding the most favorable net binding. M3/M4 gain strong gas-phase electrostatics (large negative ΔE_ele_) but these are substantially offset by large polar solvation penalties (Δ_EGB_), reducing their overall ΔG_bind_ relative to M2. Non-polar solvation (ΔE_surf_ ≈ −4 to −5 kcal·mol ⁻ ¹) modestly favors binding for all.

Fig 13 reports residue-wise MM/GBSA energy decomposition (kcal·mol ⁻ ¹) averaged over 100 snapshots from 60–100 ns; negative bars denote stabilizing contributions. Focusing on the catalytic dyad, His41 and Cys145 contribute favorably in all complexes but with distinct weights: in M2, His41 is the dominant catalytic contributor while Cys145 is modest; stabilization is further reinforced by hydrophobic residues around S2/S4 (Met49, Met165, Gln189), consistent with packing-driven binding. In M3, the profile is not dyad-centered; the most stabilizing term arises from Glu166 (S1), with substantial help from the oxyanion-loop residues Asn142/Leu141 and Met165, whereas His41 and Cys145 play secondary, yet still favorable, roles indicating an S1-anchored binding mode. In M4, both catalytic residues (His41 and Cys145) contribute strongly and comparably, alongside Met165, Glu166, and His164, revealing intimate engagement with the catalytic machinery while simultaneously exploiting S1/S2 contacts. Taken together, Fig 13 shows that dyad participation is strongest for M4, moderate for M2, and least for M3, where stabilization is instead dominated by the S1/oxyanion-loop network.

**Fig 13:**
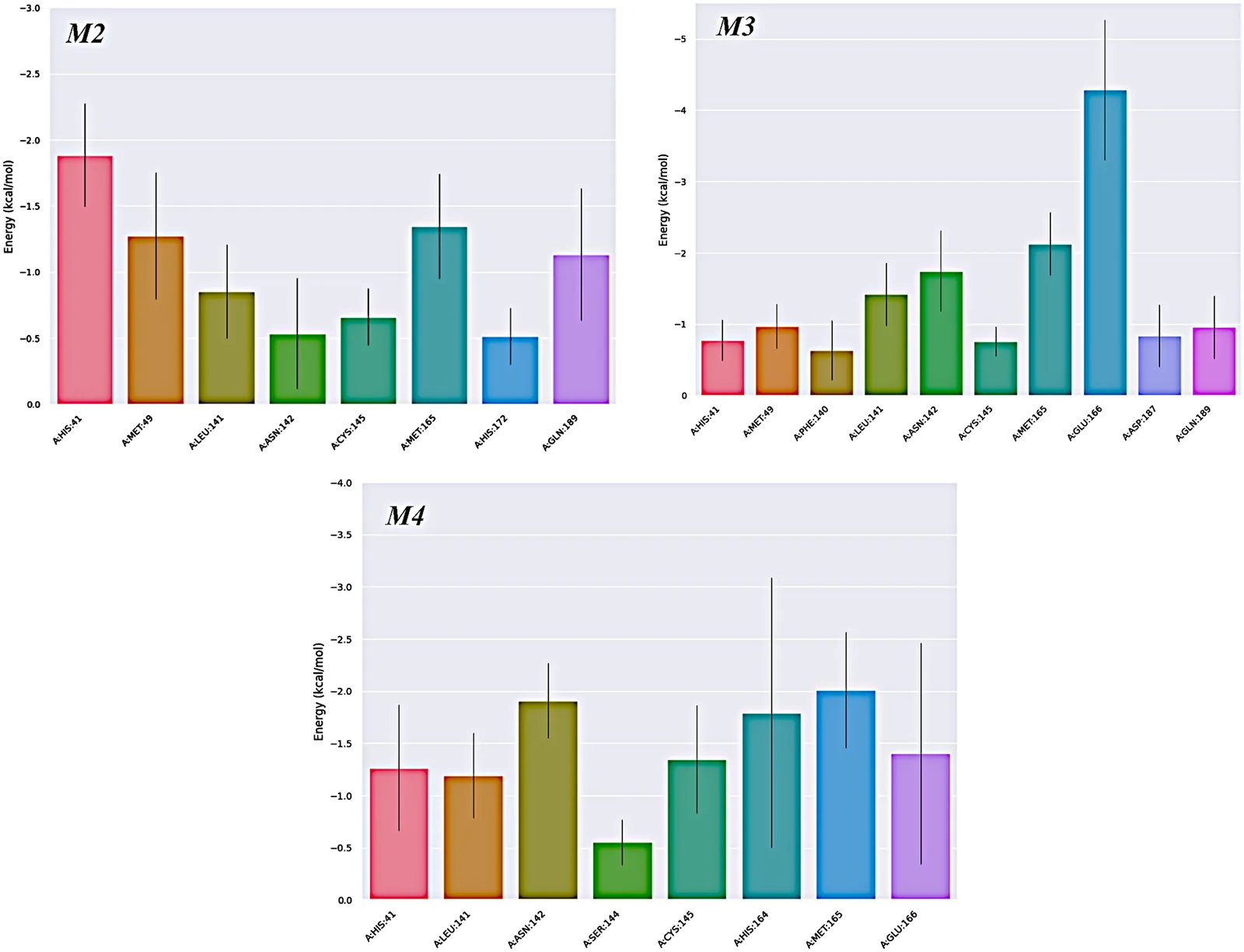
Per-residue MM/GBSA energy decomposition for M^pro^ complexes M2-M4 (kcal·mol ⁻ ¹), averaged over 100 snapshots from 60-100 ns; negative values indicate stabilizing contributions.

### 3.6 Principal-component energy landscape analysis

by reducing the thousands of degrees of freedom in an MD trajectory to a few collective modes (PC1, PC2), principal component analysis (PCA) highlights the essential motions that govern complex stability and transitions. Mapping the trajectory onto (PC1, PC2) yields a free-energy landscape (ΔG; 0–12 kJ·mol ⁻ ¹ here) where blue regions mark minima (metastable states) and red regions indicate barriers; a single, deep, localized well implies a dominant stable conformation, whereas multiple separated wells indicate interconverting substates [42].

Fig 14 presents 2D/3D landscapes for M2, M3, and M4. For M2, two low-energy minima along PC1/PC2 reveal two interconverting bound substates separated by a moderate barrier, consistent with an overall stable pose that retains local plasticity (rotamers/hydrophobic repacking). For M3, the landscape shows one elongated main basin (a blue valley) that is relatively continuous, pointing to a dominant state with coordinated fluctuations along a single collective axis coherent with S1-anchored binding (Glu166) and the high compactness observed elsewhere. For M4, a single, deep, centrally located well with steeper 3D walls indicates a tightly constrained bound conformation, compatible with intimate engagement of the catalytic dyad (His41/Cys145) together with the Glu166/Met165 network; the mild Rg “breathing” does not disrupt this dominant minimum. Altogether, Fig 14 suggests a hierarchy of conformational rigidity in the bound state M4 > M3 > M2 (one deep well for M4, one main basin for M3, two substates for M2), while confirming that all three complexes remain confined to low-energy regions during 60–100 ns sampling.

**Fig 14:**
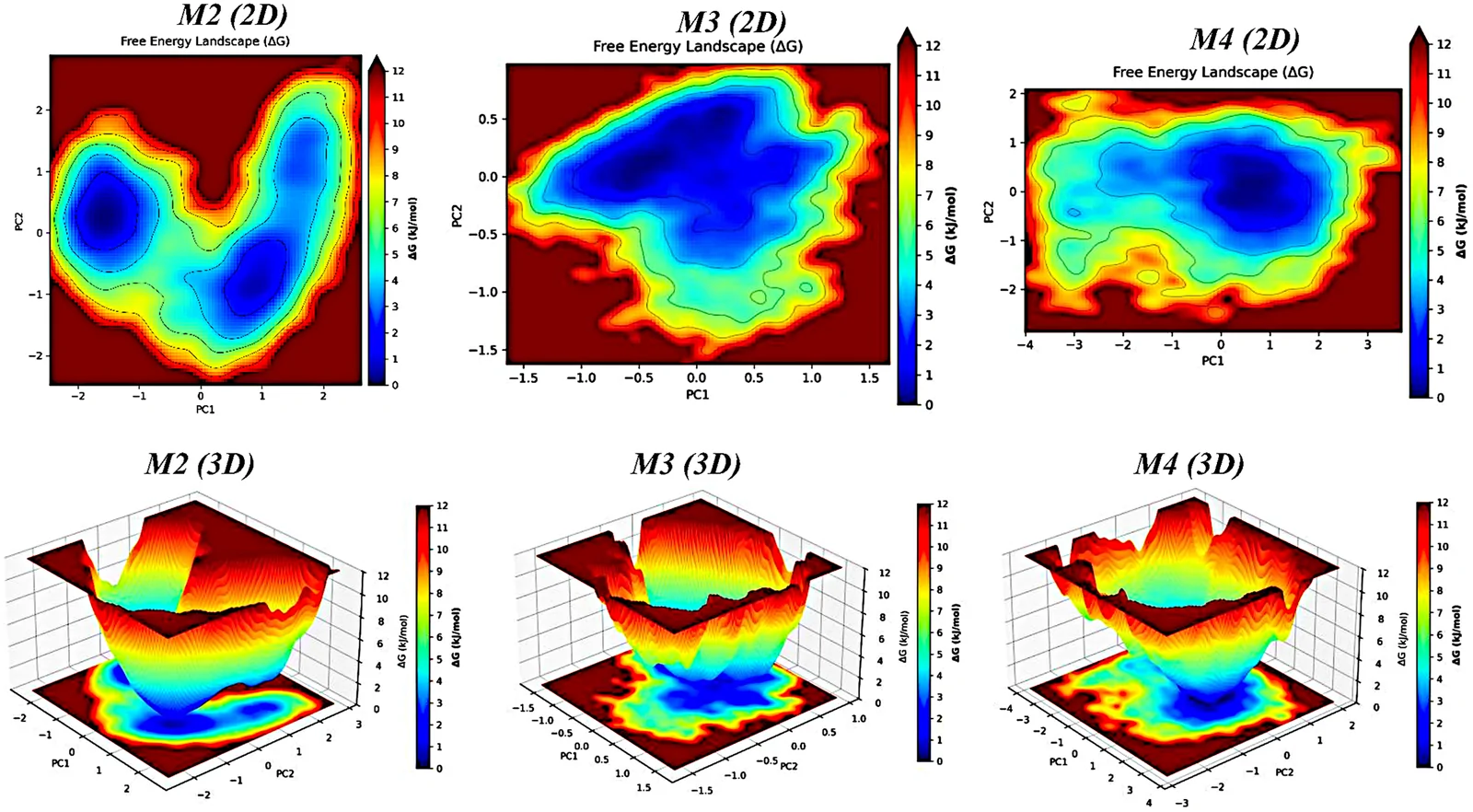
Principal component analysis (PCA) free-energy landscapes (2D/3D) for M^pro^-ligand complexes M2-M4 (kJ·mol ⁻ ¹), averaged over 60-100 ns.

### 3.7 Density Functional Theory (DFT) analysis

The DFT layer was introduced at the end of the computational workflow because docking, MD, MM/GBSA, and PCA describe how the ligands behave inside M^pro^, whereas DFT explains why the isolated chromone-tetrazole scaffolds present specific donor/acceptor, π-electronic, and weak-interaction patterns before binding [52,53]. In this context, frontier orbital energies and global descriptors are not treated as standalone potency surrogates; rather, they serve as electronic fingerprints that rationalize the trends extracted from MD and MM/GBSA, especially the effect of hydroxylation on polarity, charge redistribution, conformational preorganization, and the balance between dispersion-driven affinity and polar desolvation.

#### 3.7.1 Frontier molecular orbitals and global reactivity descriptors.

The electronic descriptors in Table 5 show that M2 and M4 share the smallest HOMO-LUMO gaps (4.00 eV) and the highest softness values (0.50 eV ⁻ ¹), whereas M3 has the largest gap (4.27 eV), highest hardness (η = 2.14 eV), and lowest softness (0.47 eV ⁻ ¹). Within conceptual DFT, these values describe relative electronic polarizability and the capacity for charge redistribution; they are not kinetic observables. Accordingly, softness cannot be used to infer association or dissociation rate constants, residence time, or covalent reactivity. M2 also has the lowest LUMO energy (−1.89 eV) and the highest electrophilicity index (ω = 3.78 eV), while M4 has the highest HOMO energy (−5.73 eV); these trends are used only as qualitative electronic context for the MD/MM/GBSA interaction patterns [44]. Direct binding kinetics and reversibility require time-dependent enzyme-inhibition and dilution-recovery experiments.

**Table 5:** Frontier orbital energies and global reactivity descriptors calculated for M2-M4 at the B3LYP-D3(BJ)/def2-SVP/CPCM(water) level.

Descriptor	M2	M3	M4
HOMO energy (eV)	−5.89	−6.08	−5.73
LUMO energy (eV)	−1.89	−1.81	−1.73
Energy gap, Egap (eV)	4.00	4.27	4.00
Hardness, η (eV)	2.00	2.14	2.00
Softness, S (eV ⁻ ¹)	0.50	0.47	0.50
Chemical potential, μ (eV)	−3.89	−3.95	−3.73
Electrophilicity index, ω (eV)	3.78	3.65	3.48

**Equations used**

Egap = E_LUMO_ - E_HOMO_

η (hardness) = Egap/ 2

S (softness) = 1/η [units: eV⁻¹]

μ (chemical potential) = (E_HOMO_ + E_LUMO_)/ 2

ω (electrophilicity) = μ²/ (2η)

The HOMO/LUMO isosurfaces in Fig 15 confirm that the frontier orbital density is not confined to a single functional group but extends over the chromone-phenyl-tetrazole axis. This electronic communication is crucial for Mpro inhibition because the chromone carbonyl/phenolic face can address the S1/oxyanion region, whereas the phenyl-tetrazole terminus maintains polar and π-mediated contacts with the entrance and S2 environment. The hydroxylation pattern finely tunes this distribution: C-3 hydroxylation in M2 reinforces the chromone polar face and increases electrophilic responsiveness, C-5 hydroxylation in M3 stabilizes the π-scaffold and increases hardness, and combined C-3/C-5 hydroxylation in M4 creates a more polarizable donor/acceptor edge suitable for direct His41/Cys145/His163/Glu166 engagement.

**Fig 15:**
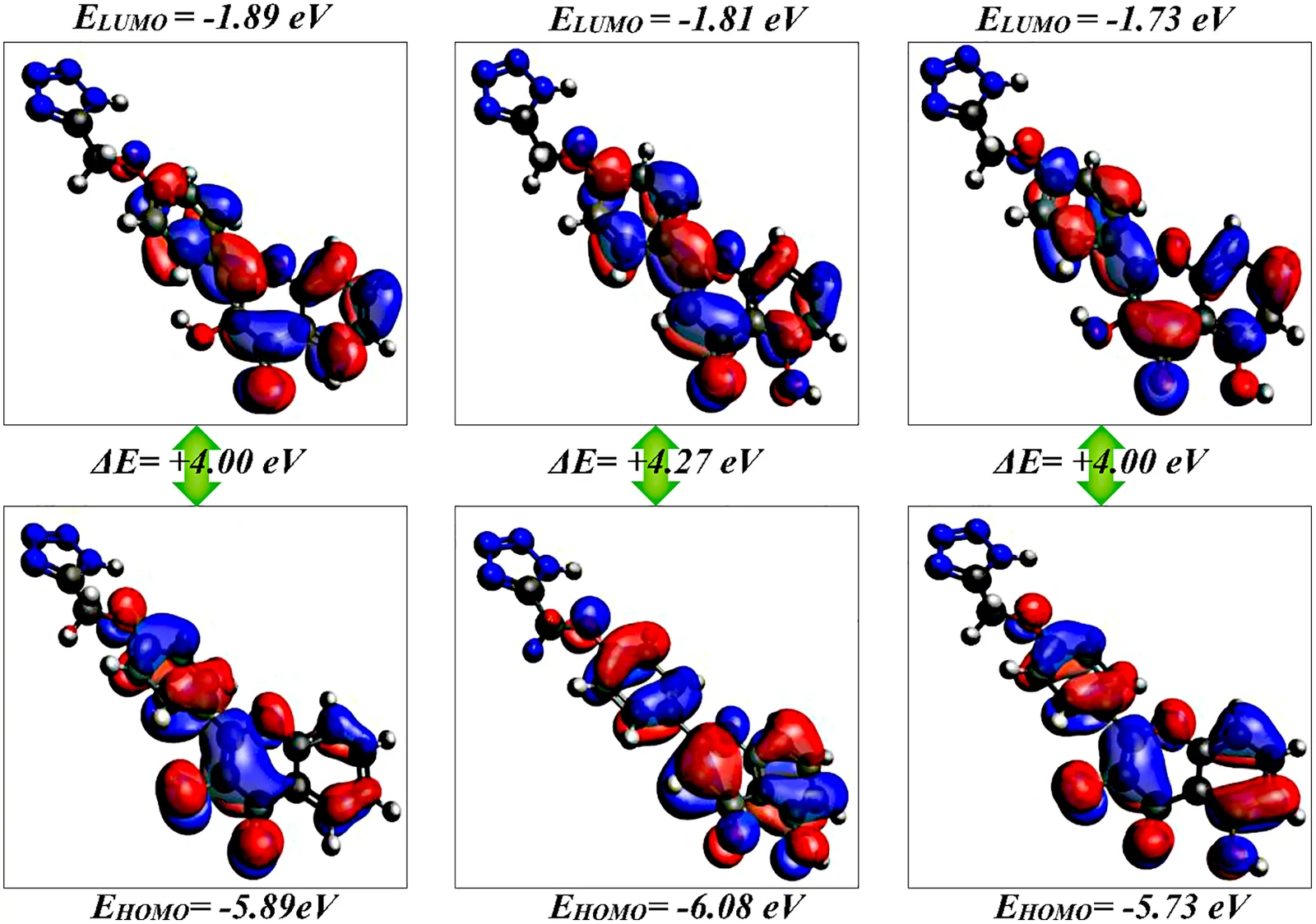
HOMO/LUMO frontier molecular orbital distributions of M2, M3, and M4 calculated at the B3LYP-D3(BJ)/def2-SVP/CPCM(water) level. The panels report the LUMO and HOMO energies and the corresponding HOMO-LUMO gaps (ΔE).

#### 3.7.2 ELF and NCI analyses of electron localization and weak intramolecular interactions.

ELF and NCI analyses were included because global descriptors alone cannot localize the electron reservoirs and weak contacts that control pharmacophoric presentation. ELF maps electron-pair localization on a 0–1 scale: high-ELF domains around heteroatoms reveal chemically available lone-pair and donor/acceptor regions, whereas more diffuse aromatic domains reveal π-platforms for hydrophobic and π-mediated contacts. NCI analysis complements ELF by mapping the reduced density gradient against sign(λ2)ρ, thereby separating attractive hydrogen-bond/dispersion regions from repulsive steric congestion. In drug-design terms, ELF identifies where the ligand can interact electronically, while NCI diagnoses whether the scaffold is internally preorganized or strained before entering the enzyme pocket [54].

The ELF maps in Fig 16 reveal intense localized domains around the chromone carbonyl oxygen, the ether/phenolic oxygen atoms, and the tetrazole nitrogens, confirming that the series exposes multiple polarizable heteroatom basins while retaining a continuous π-rich backbone. M2 displays a balanced localization pattern around the C-3 OH/carbonyl region and the tetrazole terminus, matching its dual polar anchoring and affinity-biased behavior. M3 shows a more stabilized electronic distribution and a larger HOMO-LUMO gap, consistent with a rigid S1/oxyanion-loop anchoring mode rather than a dyad-focused mode. M4 combines both hydroxyl-driven localization zones, generating a strongly anisotropic surface: a polar chromone edge for His41/Cys145/His163/Glu166 contacts and a tetrazole terminus for supplemental hydrogen bonding and π interactions. This quantum-level picture supports the PCA result for M4, where one dominant deep basin reflects a tightly constrained bound state.

**Fig 16:**
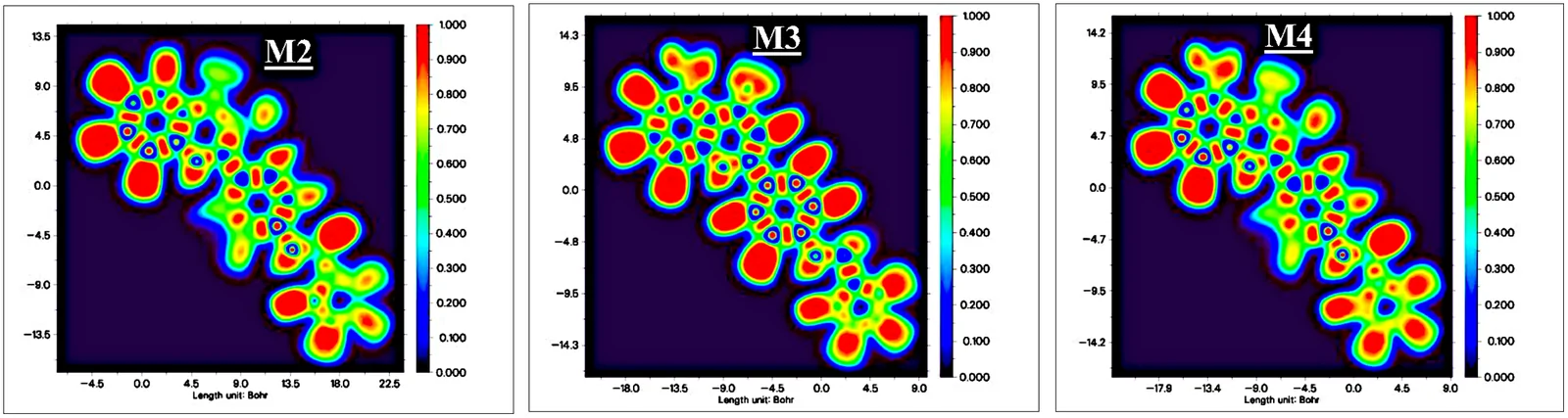
Electron localization function (ELF) maps of M2, M3, and M4, highlighting heteroatom-centered electron localization and π-delocalized regions across the chromone-phenyl-tetrazole framework.

The NCI isosurfaces and RDG plots in Fig 17 are dominated by green regions and near-zero sign(λ_2_)ρ domains, indicating that weak dispersion and intramolecular van der Waals contacts stabilize the conjugated skeleton. Local blue/cyan attractive regions near O-H···O/C = O and tetrazole-adjacent contacts indicate weak hydrogen-bond/polar preorganization, whereas limited yellow/red features reveal modest steric repulsion rather than severe strain. M2 shows sufficient weak-contact preorganization without excessive internal congestion, which is consistent with its dispersion-dominated MM/GBSA profile. M3 appears more internally stabilized and rigid, rationalizing its compact but less dyad-reactive S1 binding profile. M4 exhibits the densest polar weak-interaction network, prealigning the dihydroxychromone region for persistent catalytic-site contacts while simultaneously explaining its higher desolvation cost. Thus, ELF/NCI evidence provides a quantum rationale for the overall hierarchy: M2 is affinity-efficient, M3 is rigid and S1-anchored, and M4 is catalytically targeted.

**Fig 17:**
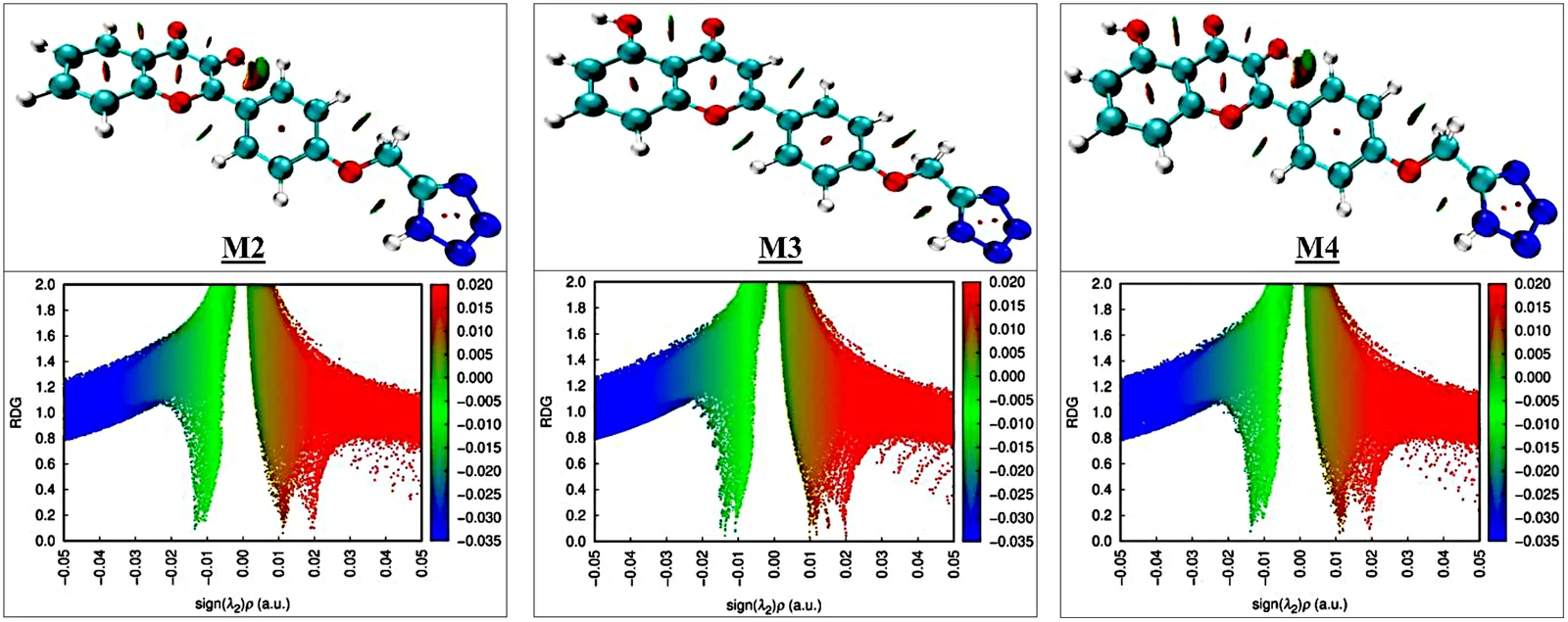
NCI isosurfaces and RDG versus sign(λ_2_)ρ scatter plots for M2-M4, showing attractive weak contacts, dispersion-stabilized zones, and limited steric repulsion within the isolated ligands.

### 3.8 Pharmacophore model derived from the integrated workflow

At the pharmacophore level, the integrated results support a provisional model comprising a planar chromone π-platform with a carbonyl/phenolic acceptor-donor edge oriented toward the S1/oxyanion region, a para-phenyl spacer for conjugation and hydrophobic occupancy, and a methoxy-tetrazole polar/π-rich head for interactions near Glu166/Phe140/His163. M2 represents an affinity-oriented version of this model, in which its electronic descriptors are consistent with dispersion/polar complementarity, whereas M4 represents a dyad-oriented version with predicted His41/Cys145 engagement.

### 3.9 Comparison with established M^pro^ inhibitor pharmacophores

Established M^pro^ pharmacophores span covalent peptidomimetic inhibitors [4,5], orally active clinical chemotypes and open-science non-covalent series [6,7], and additional non-peptidic inhibitor programs [15–18]. Covalent classes commonly combine substrate-mimetic recognition with an electrophilic group directed toward Cys145, whereas non-covalent series obtain affinity through complementary occupation of S1/S2 and polar anchoring around the Glu166/His163/Phe140 network. The present chromone-phenyl-tetrazole model shares this non-covalent S1/S2 organization but replaces a peptidomimetic backbone or reactive warhead with a planar chromone π-surface, a para-phenyl hydrophobic spacer, and a tetrazole polar head. This comparison defines a binding-mode hypothesis and does not imply equivalent potency, clinical efficacy, or selectivity.

## 4. Conclusion

This work computationally prioritizes chromone-phenyl-tetrazole hybrids as candidate non-covalent SARS-CoV-2 M^pro^ inhibitors by integrating ADMET/toxicity triage, redocking- and cross-docking-supported docking, 100-ns MD, MM/GBSA, residue-wise decomposition, PCA landscapes, and a complementary DFT/ELF/NCI quantum layer. Convergent evidence indicates stable complexes for M2-M4: M2 shows the most favorable computed ΔG_bind_ and an affinity-efficient electronic profile, M3 provides a compact and electronically hard S1-anchored state, and M4 achieves the tightest catalytic-dyad engagement through a preorganized dihydroxychromone edge. The DFT results strengthen the mechanistic interpretation by showing that M2 and M4 possess narrower HOMO-LUMO gaps and higher softness, whereas ELF/NCI maps identify heteroatom-localized electron reservoirs and weak intramolecular contacts that rationalize pharmacophoric presentation. Together, these descriptors define a compact chromone-carbonyl/phenyl/tetrazole pharmacophore that can be tuned either toward binding free-energy efficiency (M2) or direct catalytic machinery engagement (M4). Limitations include use of a single production receptor (PDB 9C8Q) despite cross-docking against three external ligand templates, the limited size of that validation panel, finite MD trajectories, no dedicated water-bridge occupancy analysis, MM/GBSA’s implicit-solvent and entropy-light approximation, prediction-based PK/toxicity and CYP classifications, use of a single neutral 1H-tetrazole microspecies without tautomeric or ionization-state sensitivity analysis, lack of human-protease selectivity counter-screening, and DFT calculations on neutral isolated ligands rather than in the fully polarizable protein environment. Experimental validation should begin with a purified M^pro^ FRET cleavage assay to determine IC_50_ values, followed by full kinetic analysis for Ki and inhibition mode. Time-dependent inhibition and jump-dilution or dilution-recovery experiments should test the predicted reversible non-covalent mechanism. Orthogonal target-engagement methods such as differential scanning fluorimetry or native mass spectrometry, followed by antiviral efficacy and CC_50_ measurements in a permissive cell model, would establish target engagement, cellular activity, and selectivity index. Solubility, permeability, microsomal stability, and CYP3A4-inhibition assays should be performed in parallel; co-crystallography or cryo-EM would provide definitive structural confirmation of the predicted S1/S2/dyad contacts. Overall, M2 and M4 emerge as complementary lead chemotypes for experimental validation and structure-guided optimization.

## Supporting information

S1 Fig Structural superposition of the SARS-CoV-2 Mpro receptor templates used for cross-docking validation. Chain A Cα atoms of 6M2N, 6W63, and 7VU6 were aligned to the 9C8Q reference frame, yielding refined Cα RMSDs of 0.425, 0.305, and 0.510 Å, respectively. The co-crystallized ligands were transformed with their receptors and retained as experimental reference poses for subsequent ligand RMSD calculation.
(DOCX)

S2 Fig Comparative docking of M1-M4 in the active-site cavity of human CYP3A4 (PDB 3NXU). Two-dimensional interaction maps and AutoDock Vina binding scores are shown for M1 (−9.5), M2 (−9.0), M3 (−9.0), and M4 (−8.8 kcal mol-1). The heme cofactor was retained during receptor preparation. These results indicate active-site compatibility but are not interpreted as quantitative evidence of CYP3A4 inhibition or as a direct decomposition of steric and electronic effects.
(DOCX)

S1 Table Cross-docking validation of external ligands against the 9C8Q receptor. (DOCX)

S1 Method Comparative molecular docking against human CYP3A4. (DOCX)
