# Supplementary material for: Computational evaluation of chromone–tetrazole hybrids as noncovalent inhibitors of SARS-CoV-2 Mpro
Source: PLoS One. 2026 Nov 11;21(11):e0362385. doi: 10.1371/journal.pone.0362385 (PMC13754155; doi:10.1371/journal.pone.0362385)
Supplement: Supplementary file 1 [file pone.0362385.s001.docx]

**Refined Cα RMSD: 0.425 Å**

**9C8Q**

**6M2N**

**Refined Cα RMSD: 0.305 Å**

**9C8Q**

**6W63**

**Refined Cα RMSD: 0.510 Å**

**9C8Q**

**7VU6**

**Figure S1.** Structural superposition of the SARS-CoV-2 Mpro receptor templates used for cross-docking validation. Chain A Cα atoms of 6M2N, 6W63, and 7VU6 were aligned to the 9C8Q reference frame, yielding refined Cα RMSDs of 0.425, 0.305, and 0.510 Å, respectively. The co-crystallized ligands were transformed with their receptors and retained as experimental reference poses for subsequent ligand RMSD calculation.
