# Supplementary material for: Computational evaluation of chromone–tetrazole hybrids as noncovalent inhibitors of SARS-CoV-2 Mpro
Source: PLoS One. 2026 Nov 11;21(11):e0362385. doi: 10.1371/journal.pone.0362385 (PMC13754155; doi:10.1371/journal.pone.0362385)
Supplement: Supplementary file 2 [file pone.0362385.s002.docx]

**M1-3NXU**

**M2-3NXU**

**M3-3NXU**

**M4-3NXU**

**-9.5 kcal mol⁻¹**

**-9.0 kcal mol⁻¹**

**-9.0 kcal mol⁻¹**

**-8.8 kcal mol⁻¹**

**Figure S2.** Comparative docking of M1-M4 in the active-site cavity of human CYP3A4 (PDB 3NXU). Two-dimensional interaction maps and AutoDock Vina binding scores are shown for M1 (-9.5), M2 (-9.0), M3 (-9.0), and M4 (-8.8 kcal mol-1). The heme cofactor was retained during receptor preparation. These results indicate active-site compatibility but are not interpreted as quantitative evidence of CYP3A4 inhibition or as a direct decomposition of steric and electronic effects.
