# Supplementary material for: Computational evaluation of chromone–tetrazole hybrids as noncovalent inhibitors of SARS-CoV-2 Mpro
Source: PLoS One. 2026 Nov 11;21(11):e0362385. doi: 10.1371/journal.pone.0362385 (PMC13754155; doi:10.1371/journal.pone.0362385)
Supplement: Supplementary file 3 [file pone.0362385.s003.docx]

**Table S1.** Cross-docking validation of external ligands against the 9C8Q receptor.

| **Ligand** | **Native experimental structure** | **Docking receptor** | **Validation type** | **RMSD (Å)** |
| --- | --- | --- | --- | --- |
| **Baicalein** | 6M2N | 9C8Q | Cross-docking | 0.745 |
| **X77** | 6W63 | 9C8Q | Cross-docking | 0.128 |
| **Ensitrelvir** | 7VU6 | 9C8Q | Cross-docking | 1.473 |
| **A1AU4** | 9C8Q | 9C8Q | Redocking | 0.30 |
