# Supplementary material for: Computational evaluation of chromone–tetrazole hybrids as noncovalent inhibitors of SARS-CoV-2 Mpro
Source: PLoS One. 2026 Nov 11;21(11):e0362385. doi: 10.1371/journal.pone.0362385 (PMC13754155; doi:10.1371/journal.pone.0362385)
Supplement: Supplementary file 4 [file pone.0362385.s004.docx]

**S1 Method. Comparative molecular docking against human CYP3A4**

To examine whether the pkCSM CYP3A4 classifications were compatible with active-site accommodation, an exploratory comparative docking analysis was performed in PyRx using AutoDock Vina and AutoDock Tools (ADT v1.5.7). The 2.00 Å crystal structure of human CYP3A4 bound to ritonavir (PDB 3NXU) was used, with chain A retained as the receptor. Crystallographic water molecules and nonessential heteroatoms were removed, whereas the catalytic heme cofactor was preserved. Polar hydrogens and Gasteiger charges were added, and the receptor was converted to PDBQT format. M1-M4 and ritonavir were energy-minimized and converted to PDBQT using the same ligand-preparation workflow as in the principal Mpro docking analysis.

Docking was restricted to a search box centered on the ritonavir-defined CYP3A4 active-site cavity and encompassing the heme-adjacent binding pocket. AutoDock Vina exhaustiveness was set to 8; all remaining parameters were kept at their default values and identical for all ligands. Protocol performance was assessed by redocking ritonavir; the top-ranked pose reproduced the crystallographic orientation with a ligand heavy-atom RMSD of 0.708 Å, supporting geometric reproducibility of the selected pocket and grid. M1-M4 were then docked with the validated settings, and the top-ranked pose of each compound was inspected in Discovery Studio. The resulting Vina scores and interaction maps are shown in Figure S2.

This analysis was conducted solely as a structural comparison to interrogate the pkCSM classification. Because docking estimates active-site compatibility rather than enzyme inhibition, and because the score differences were small, the results were not used to assign CYP3A4 inhibitory potency or to separate steric from electronic contributions. The neutral 1H-tetrazole input state used in the Mpro calculations was retained for consistency; no alternative neutral 2H tautomer or deprotonated tetrazolate was evaluated, so this comparative CYP3A4 analysis is likewise conditional on that assignment.
